# Biofertilizers as Strategies to Improve Photosynthetic Apparatus, Growth, and Drought Stress Tolerance in the Date Palm

**DOI:** 10.3389/fpls.2020.516818

**Published:** 2020-10-23

**Authors:** Mohamed Anli, Marouane Baslam, Abdelilah Tahiri, Anas Raklami, Sarah Symanczik, Abderrahim Boutasknit, Mohamed Ait-El-Mokhtar, Raja Ben-Laouane, Salma Toubali, Youssef Ait Rahou, Mustapha Ait Chitt, Khalid Oufdou, Toshiaki Mitsui, Mohamed Hafidi, Abdelilah Meddich

**Affiliations:** ^1^Laboratory of Agro-Food, Biotechnologies and Valorization of Plant Bioresources (AGROBIOVAL), Department of Biology, Faculty of Science Semlalia, Cadi Ayyad University (UCA), Marrakesh, Morocco; ^2^Laboratory of Microbial Biotechnologies, Agrosciences, and Environment (BioMAgE), Faculty of Science Semlalia, Cadi Ayyad University (UCA), Marrakesh, Morocco; ^3^Laboratory of Biochemistry, Faculty of Agriculture, Niigata University, Niigata, Japan; ^4^Department of Soil Sciences, Research Institute of Organic Agriculture Frick (FiBL), Frick, Switzerland; ^5^Domaines Agricoles, Laboratoire El Bassatine, Domaine El Bassatine, Meknès, Morocco; ^6^Mohammed VI Polytechnic University (UM6P), Agrobiosciences program (AgBs), Benguerir, Morocco

**Keywords:** arbuscular mycorrhizal fungi, climate change, compost, PGPR, plant fitness, photosynthesis, agro-physiological responses, water deficit

## Abstract

Rainfall regimes are expected to shift on a regional scale as the water cycle intensifies in a warmer climate, resulting in greater extremes in dry versus wet conditions. Such changes are having a strong impact on the agro-physiological functioning of plants that scale up to influence interactions between plants and microorganisms and hence ecosystems. In (semi)-arid ecosystems, the date palm (*Phoenix dactylifera* L.) -an irreplaceable tree- plays important socio-economic roles. In the current study, we implemeted an adapted management program to improve date palm development and its tolerance to water deficit by using single or multiple combinations of exotic and native arbuscular mycorrhizal fungi (AMF1 and AMF2 respectively), and/or selected consortia of plant growth-promoting rhizobacteria (PGPR: B1 and B2), and/or composts from grasses and green waste (C1 and C2, respectively). We analyzed the potential for physiological functioning (photosynthesis, water status, osmolytes, mineral nutrition) to evolve in response to drought since this will be a key indicator of plant resilience in future environments. As result, under water deficit, the selected biofertilizers enhanced plant growth, leaf water potential, and electrical conductivity parameters. Further, the dual-inoculation of AMF/PGPR amended with composts alone or in combination boosted the biomass under water deficit conditions to a greater extent than in non-inoculated and/or non-amended plants. Both single and dual biofertilizers improved physiological parameters by elevating stomatal conductance, photosynthetic pigments (chlorophyll and carotenoids content), and photosynthetic efficiency. The dual inoculation and compost significantly enhanced, especially under drought stress, the concentrations of sugar and protein content, and antioxidant enzymes (polyphenoloxidase and peroxidase) activities as a defense strategy as compared with controls. Under water stress, we demonstrated that phosphorus was improved in the inoculated and amended plants alone or in combination in leaves (AMF2: 807%, AMF1+B2: 657%, AMF2+C1+B2: 500%, AMF2+C2: 478%, AMF1: 423%) and soil (AMF2: 397%, AMF1+B2: 322%, AMF2+C1+B2: 303%, AMF1: 190%, C1: 188%) in comparison with controls under severe water stress conditions. We summarize the extent to which the dual and multiple combinations of microorganisms can overcome challenges related to drought by enhancing plant physiological responses.

## Introduction

Owing to rapid climate change, drought is becoming one of the most important environmental stresses that is outside of plants’ physiological limits and is causing a substantial decline in crop productivity (Shaw and Etterson, 2012; Sarwat and Tuteja, 2017). Drought influences transport and availability of soil nutrients (Vurukonda et al., 2016), and it affects morphological, physiological, and nutritional traits of plants especially water content, leaf water potential, photosynthetic pigment, stomatal conductance and phosphorus (P) and nitrogen (N) absorption (Jaleel et al., 2009; Augé et al., 2014; Baslam et al., 2014; Meddich et al., 2015a, 2018; Symanczik et al., 2018). Drought also affects antioxidant defense leading to oxidative stress owing to the accumulation of reactive oxygen species (ROS) such as hydrogen peroxide (Maheshwari et al., 2012; Vurukonda et al., 2016; Abdel Latef et al., 2019a,b). On a higher scale, when plants are exposed to drought that alters their photosynthesis machinery, this can shift source/sink relationships of photosynthate, symbiotic interactions, plant growth, and fitness (Becklin et al., 2016; Sever et al., 2018). The inhibition and degradation of chlorophyll synthesis through ROS over-accumulation leads to a decrease of photosynthetic activity (Abd El-Mageed et al., 2018; Duo et al., 2018).

The date palm (*Phoenix dactylifera* L.) is a dioecious evergreen tree with major ecological and socio-economic roles in many countries, mainly arid zones (Chao and Krueger, 2007). Indeed, the economic importance of the palm family of plants (Arecaceae) ranks second only to the grass family (Poaceae) among monocotyledons and the third in the world (after the Gramineae and Leguminosae families) (FAO^1^). The economic utility of these palms is based on their fruits and derived beverages as a staple food, their wood, as palms are used in building and the artisanal sector, where they are used for ornamental purposes (Arias et al., 2016). In addition, in oasis ecosystems, date palms create a microclimate that is essential for the cultivation of underlying crops such as fruit trees, vegetables and forage species (Meddich et al., 2019). However, date palm groves are subjected to biotic (primary *fusarium* wilt) and abiotic (mainly drought and salinity) constraints as well as low soil fertility and management that have decimated this crop thus deteriorating oasis ecosystems, hindering agricultural production under this environmental instability, and driving higher levels of migration (Oihabi, 1991; Meddich et al., 2015a, 2018; Arias et al., 2016; Meddich and Boumezzough, 2017; FAO, 2018; Whitman, 2019). Factors such as drought, exacerbated by climate change, will affect plant physiology by altering plant-organism interactions since plant species evolve in complex environments with networks of interacting species. Although studies have examined various ways of handling stress inducers to increase plant survival and performance, little has been done on the integrative aspect of this improvement approach to protecting and empowering plants to resist and grow better under drought conditions. In this regard, management practices and strategies that allow plants to resist abiotic and biotic stresses are urgently required and should be exploited to improve agricultural production (Meddich et al., 2018, 2019), reduce the use of pesticides and chemical fertilizers, and protect crops and soil quality (Shen et al., 2013). It should be noted that the plant nursery industry –being trees and fruit plants the most important nursery crops- constitutes a large and growing sector of agriculture. The regular practice of cultivating vitro plants by farmers under nursery conditions prior to field transplantation involves growing the plants only in sandy soil without any amendments. At the time of planting is especially apparent when outplants face stresses and/or strong competition from other vegetation. At this stage, the growth potential of planting stock plays a primordial role for the transplanting success. The application of biofertilizers such as organic fertilizers and beneficial soil microorganisms has emerged as a potential solution to promote plant fitness, boost yield, and tolerance to environmental constraints (Abdel Latef and Chaoxing, 2011; Meddich et al., 2015b; Abdel Latef et al., 2016; Padilla et al., 2017; Júnior et al., 2018; Symanczik et al., 2018; Ait-El-Mokhtar et al., 2019; Ben-Laouane et al., 2019; Raklami et al., 2019). The appropriate management of plant nutrition, growth, and tolerance to drastic constraints such as drought, salinity and soil poverty is becoming a key component in increasing crop yield under changing environmental conditions (Zou and Wu, 2011; Baslam and Goicoechea, 2012; Maheshwari et al., 2012; Hidri et al., 2016; De Pascale et al., 2017; Meddich et al., 2018). In addition to regulating nutrient acquisition, inoculation with Arbuscular Mycorrhizal Fungi (AMF) and Plant Growth Promoting Rhizobacteria (PGPR) are successfully being seen to be an effective substitute to ensure stable, safe and sustainable agricultural and biomass production (Wu and Zou, 2017; Zhang et al., 2018; Kumar et al., 2019; Kumari et al., 2019; Abdel Latef et al., 2020). Indeed, the beneficial effects of the inoculation by the rhizosphere microbial communities are linked with nutrient recycling, mineral nutrition. solubilisation of nutrients such as P, potassium (K) and iron (Fe), biodegradation of soil organic matter, phytohormone and antibiotics productions, improvements in soil structure and aggregation, and enhancement of plant resistance to pests and diseases (Al-Karaki, 2000, 2006; Al-Karaki et al., 2004; Nadeem et al., 2014; Rillig et al., 2014; Grobelak et al., 2015; Kumar et al., 2015; Meddich et al., 2018, 2019; Symanczik et al., 2018; Raklami et al., 2019; Wu et al., 2019). The (co)-inoculants of PGPR and/or AMF can advance the nutrient use efficiency of fertilizers (Adesemoye et al., 2009). Moreover, AMF and/or PGPR inoculation could mitigate the detrimental effect of stresses through the enhancement of photosynthetic efficiency, overproduction of antioxidant enzymes and/or non-enzymatic antioxidants, and/or activation of the mycorrhizal induced resistance (MIR) mechanism by bypassing plant defense (Pozo and Azcón-Aguilar, 2007; Bompadre et al., 2014; Nadeem et al., 2014; Pieterse et al., 2014; Gusain et al., 2015; Lenoir et al., 2016; Duo et al., 2018; Javan Gholiloo et al., 2019). Although PGPR and AMF are commonly applied as beneficial microorganisms in agriculture and several studies have been devoted to studying the influence of this symbiosis on the plant response to abiotic stress, the mechanisms responsible for increased plant tolerance to stress have yet to be fully elucidated. Also, little is known about the agro-physiological changes occurring in the plants when these microorganisms are applied together. It has been suggested that the combined application of both microorganisms has positive effects on the nutritional composition of several plant species (Nadeem et al., 2014; Ben-Laouane et al., 2019).

Furthermore, the exogenous introduction of beneficial organic substances such as compost has been evaluated previously (Nikitas et al., 2008) as a soil conditioner in agriculture or a substitute to chemical fertilizers to improve resilience, yield, and tolerance of plants to the toxicity of these stress-imposed conditions. The use of local composts represents an eco-friendly alternative for plant growth, mineral nutrition, soil organic matter content, and soil properties such as water retention capacity and soil suppressiveness (Shen et al., 2013; Luciens et al., 2014; Mehta et al., 2014; Kwey et al., 2015; Ning et al., 2017). Previous studies have suggested that compost application in soil increased the supply of organic carbon and N for microbial communities and improved soil health and plant yield (Shen et al., 2013; Liu et al., 2018). Further, compost application triggers plant resistance to different environmental stresses (Tartoura et al., 2014; Ortuño et al., 2018) by increasing the photosynthetic activity (Abd El-Mageed et al., 2019; Khosravi Shakib et al., 2019). In the current research, we explored the agro-physiological and biochemical responses involved in drought adaptation in date palms, and the functionality of the single and dual-use of selected strains of PGPR and native and exotic AMF with or without the addition of two composts. The objective of this study was to evaluate the morpho-physiological basis of drought responses in date palms under implementation of eco-friendly cultivation practices. The results obtained here will provide a deeper understanding of the mechanisms of date palm tolerance to long-term drought stress as well as paving the way for identification of the best factors that led to successful outcomes in the biofertilization experiments for other crops.

## Materials and Methods

### Biofertilizer Materials

Two types of AMF inoculants were used in our experiment: (i) an exogenous AMF strain (*Rhizoglomus irregulare*, DAOM 197198) provided by the Plant Biotechnology Institute of Montreal (Canada) and (ii) an indigenous consortium of AMF isolated from the Tafilalet palm grove located 500 km southeast of Marrakesh and containing a mixture of native species: (i) *Glomus* sp. (15 spores/g of substrate), (ii) *Sclerocystis* sp. (9 spores/g substrate), and (iii) *Acaulospora* sp. (1 spore/g of substrate) (Meddich et al., 2015a). The inoculum was enriched in propagules by co-cultivation with *Zea mays* L. as the host plant under controlled greenhouse conditions. Corn roots containing hyphae, vesicles, and spores were harvested, cut into small pieces and used as the inoculum. Inoculation of date palm was performed by adding 40 g of the inoculum (roots and substrate containing spores) to the date palm root system. Non-mycorrhizal (NM) treatments received an equal quantity of both non-inoculated (and non-mycorrhizal) *Z. mays* roots to match ‘organic matter’ in the pots and filtered inoculum in an attempt to restore other soil free-living microorganisms accompanying the AMF. The filtrate for each pot was obtained by passing the mycorrhizal inoculum in 20 mL of distilled water through a layer of 15- to 20-μm filter papers (Whatman, GE Healthcare, Buckinghamshire, United Kingdom).

The bacterial inocula used in our study consisted of four PGPR isolates (Z1, Z2, Z4, and ER21 strains) isolated from the date palm groves rhizosphere (Tafilalet, Morocco). The inocula were prepared by growing the strains in Tryptic Soy Broth (TSB) liquid culture at 28°C to an optical density of 1 at 600 nm (about 10^9^ CFU/mL). The plant inoculations were carried out by adding 4 mL of the bacterial suspension formed from the four abovementioned strains into equal volumes closer to the roots. After 15 days, a second inoculation (booster) was carried out by placing another 4 mL of the bacterial suspension next to the plant roots to increase the bacterial rate in the soil and ensure the infection of the newly formed roots.

The quantification *in vitro* of plant growth-promoting traits of the strains used was examined by standard protocols: phosphate solubilization was performed by the production of halo on agar medium as described by Alikhani et al. (2006) and the tolerance to water deficiency was tested by the resistance to polyethylene glycol. A confrontation assay was carried out to confirm the absence of inhibition between the four strains. The PGPR characteristics of the four strains are listed in Table 1.

**TABLE 1:** Phosphate solubilization and resistance to polyethylene glycol (tolerance to water deficiency) of the four tested PGPR strains (Z1, Z2, Z4, and ER21).

Activity	Z1	Z2	Z4	ER21
Phosphate solubilization	+	+	+	+
Resistance to polyethylene glycol 6000	+	+	−	−

The composts used were prepared from grass (C1) and a mixture of green (C2) waste as described by Meddich et al. (2016). The composts (5% *W*/*W* with respect to culture soil) were added to the corresponding pots at date palm vitroplants transplanting (Anli et al., 2020). The physicochemical and microbiological properties of the two composts are presented in Table 2.

**TABLE 2:** Physico-chemical and microbiological properties of the composts used in this study.

Composts	pH	EC (mS/cm)	COT (%)	NTK (%)	C/N	P (mg/g)	Bacterial population (CFU/g)	Fungal population (CFU/g)
Compost (C1)	7.86	7.10	30.65	2.19	14.00	0.270	1.65 × 10^8^	4.30 × 10^5^
Compost (C2)	7.80	8.50	27.24	1.32	20.64	0.266	2.12 × 10^5^	9.75 × 10^4^

*EC, electrical conductivity; TOC, total organic carbon; TKN, total Kjeldahl-nitrogen; C/N, carbon-to-nitrogen ratio; P, phosphorous; CFU, colony-forming unit.*

### Experimental Design

Date palm vitroplants (*Phoenix dactylifera* L.) of variety Boufgouss -an elite variety with high commercial importance- were transplanted at the two leaf stage into 2.4 L plastic buckets filled (4/5) with sterilized soil (at 180°C for 3 h on 3 consecutive days) alone or mixed with compost at 5%. The soil used (bulk density: 1.32 ± 0.01 g cm^–3^) had the following characteristics: sand, 51%; clay, 19%; loam, 30%, available phosphorus, 11 ppm; organic matter, 1%; total organic carbon, 0.58%; nitrogen, 0.84 mg/g; EC 0.19 mS/cm; and pH, 8.6. The soil bulk density did not vary in every treatment. The finely textured soil to successfully grow date palm, instead of sand, has been used to achieve fast equilibrium rates during drying. The plants were watered and maintained at 75% field capacity (FC). During the pre-sowing period, irrigation was applied to FC to ensure full stand establishment in all treatments. Soil moisture was measured randomly in untreated and treated pots in each treatment using a TDR meter (Delta UK Ltd., Clacton-on-Sea, United Kingdom) in the morning and evening of each day. According to the measured soil water content, soil bulk density, soil moisture maximum field capacity and soil weight, the amount of needed water under different water conditions was calculated. Plants were grown in the greenhouse at 25.5°C (16/8 h light/dark) with fluorescent lighting (500 μmol m^–2^ s^–1^) and average relative humidity of 68.5%.

The experiment was carried out in a fully randomized design with 10 biological replicates for each treatment (in total 54 treatments) and all plants were placed randomly in the greenhouse (Table 3).

**TABLE 3:** Different treatments (and their nomenclature) applied in this study.

Treatments	Water regime	
Control	75% FC	Plants non-amended with compost and no-inoculated with AMF/PGPR
B1	25% FC	Plants non-amended with compost, no-inoculated with AMF, and inoculated with PGPR consortia B1 (Z1+Z2)
B2		Plants non-amended with compost, no-inoculated with AMF, and inoculated with PGPR consortia B2 (Z1+Z2+Z4+ER21)
C1		Plants amended with compost C1 (grass waste), no-inoculated with AMF/PGPR
C1 + B1		Plants amended with compost C1, no-inoculated with AMF, and inoculated with PGPR consortia B1
C1 + B2		Plants amended with compost C1. no-inoculated with AMF, and inoculated with PGPR consortia B2
C2		Plants amended with compost C2 (mixture of green waste) and no-inoculated with AMF/PGPR
C2 + B1		Plants amended with compost C2, no-inoculated with AMF, and inoculated with PGPR consortia B1
C2 + B2		Plants amended with compost C2. no-inoculated with AMF and inoculated with PGPR consortia B2
AMF1		Plants inoculated with AMF1 (exogenous *R. irregulare*), non-amended with compost, and no-inoculated with PGPR
AMF1 + B1		Plants inoculated with AMF1, inoculated with PGPR consortia B1, and non-amended with compost
AMF1 + B2		Plants inoculated with AMF1, inoculated with PGPR consortia B2, and non-amended with compost
AMF1 + C1		Plants inoculated with AMF1, amended with C1, and no-inoculated with PGPR
AMF1 + C1 + B1		Plants inoculated with AMF1, inoculated with PGPR consortia B1, and amended with C1
AMF1 + C1 + B2		Plants inoculated with AMF1, inoculated with PGPR consortia B2, and amended with C1
AMF1 + C2		Plants inoculated with AMF1, amended with C2, and no-inoculated with PGPR
AMF1 + C2 + B1		Plants inoculated with AMF1, inoculated with PGPR consortia B1, and amended with C2
AMF1 + C2 + B2		Plants inoculated with AMF1, inoculated with PGPR consortia B2, and amended with C2
AMF2		Plants inoculated with AMF2 (indigenous consortium of AMF), non-amended with compost, and no-inoculated with PGPR
AMF2 + B1		Plants inoculated with AMF2, inoculated with PGPR consortia B1, and non-amended with compost
AMF2 + B2		Plants inoculated with AMF2, inoculated with PGPR consortia B2, and non-amended with compost
AMF2 + C1		Plants inoculated with AMF2, amended with C1, and no-inoculated with PGPR
AMF2 + C1 + B1		Plants inoculated with AMF2, inoculated with PGPR consortia B1, and amended with C1
AMF2 + C1 + B2		Plants inoculated with AMF2, inoculated with PGPR consortia B2, and amended with C1
AMF2 + C2		Plants inoculated with AMF2, amended with C2, and no-inoculated with PGPR
AMF2 + C2 + B1		Plants inoculated with AMF2, inoculated with PGPR consortia B1, and amended with C2
AMF2 + C2 + B2		Plants inoculated with AMF2, inoculated with PGPR consortia B2, and amended with C2

Three months after experiment start, two water regimes were imposed 75 and 25% FC (Baslam et al., 2014; Meddich et al., 2015a, 2018).

### Chlorophyll Fluorescence and Stomatal Conductance Determinations

Chlorophyll fluorescence was measured by a fluorometer (OPTI-SCIENCE, OS30p). Dark adaptation was made on the upper side of the second fully developed leaf from the apex by obscuring for 20 min. This parameter was measured by transmission at 650 nm on a leaf area of 12.5 mm^2^. The fluorescence signal was recorded for a second at an acquisition speed of 10 μs (Strasser and Strasser, 1995). Stomatal conductance (g_*s*_) was determined as described by Harley et al. (1992).

### Photosynthetic Pigments Quantification

The concentration of chlorophyll *a*, *b*, total chlorophyll, and carotenoids was determined according to the method described by Arnon (1949). Photosynthetic pigments were extracted from the frozen leaf powder subsample using cold acetone 80%. Following centrifugation at 10,000 × *g* for 10 min, supernatant absorbance was read at 480, 645, and 663 nm using a UV–visible spectrophotometer (UV-3100PC spectrophotometer, VWR).

### Leaf Water Potential

Leaf water potential (Ψ_w_) was measured using a pressure chamber (Model 600-EXP Super Pressure Chamber, PMS instrument, Albany, OR, United States) at predawn (06:00–08:00 h). The measurements were taken on mature fully expanded leaves from the upper part of the stem. Cutting leaves water potential were measured over the same days and immediately after gas exchange measurements.

### Growth Assessment and Mineral Analysis

The growth performance of date palm plants was assessed by measuring the number of leaves, shoot height, root length, leaf area, and total dry matters (DM; obtained after drying samples at 80°C until the weight remained constant). The first fully expanded leaf of date palms in each treatments was harvested at the end of the light period, snap-frozen, ground to a fine powder in liquid N using a pestle and mortar, and kept at −80°C for the subsequent biochemical analyses.

For mineral analyses, the dried shoots were grounded using a coffee mill. Shoot N concentration was measured according to the method described by Rodier (1984). Shoot P concentration was estimated using the Olsen method (Olsen and Sommers, 1982) by incinerating the shoot powder (500 mg) in a muffle furnace before acid extraction.

### Mycorrhization Assessment

Root samples were washed with distilled water and cleaned with 10% of KOH at 90°C for 30 min. Then, they were washed again and acidified with 2% HCl for 10 min and stained with Trypan blue at 90°C for 20 min according to Phillips and Hayman (1970). The microscopic assessment of mycorrhizal root colonization rates was performed according to the method of Trouvelot et al. (1986).

### Total Soluble Sugars Quantification

Total soluble sugars (TSS) were determined in 0.1 g of the frozen leaf powder in 80% (v/v) ethanol. The quantity of TSS was determined according to Dubois et al. (1956) in 0.2 mL of the supernatant mixed with 0.2 mL of phenol and 1 mL of concentrated sulfuric acid. After 15 min, TSS content was determined by measuring the absorbance at 485 nm and calculated using the standard glucose curve.

### Total Soluble Proteins and Antioxidant Enzymes

Frozen leaf powder subsamples (0.1 g) were homogenized in a cold mortar with 4 mL of 1 M phosphate buffer (pH 7) containing 5% polyvinylpolypyrrolidone (PVPP). The homogenate was centrifuged at 18,000 × *g* for 15 min at 4°C and the supernatant was used to measure antioxidant enzyme activities (Tejera García et al., 2004). Total soluble proteins were determined according to the technique described by Bradford (1976). Peroxidase (POX) activity was measured as described previously (Hori et al., 1997). The reaction mixture (3 mL) contained 1 M phosphate buffer (pH 7.0), 20 mM guaiacol, 40 mM H_2_O_2_, and 0.1 mL of the enzymatic extract which was added to start the reaction. POX activity was determined at 470 nm by its ability to convert guaiacol to tetraguaiacol (ε = 26.6 mM^–1^⋅cm^–1^) One unit of POX activity was defined as an absorbance change of 0.01 unit min^–1^. Polyphenol oxidase (PPO) was estimated by the method of Hori et al. (1997). The assay solution contained 20 mM catechol in 0.1 M phosphate buffer (pH 7). The reaction was started by addition of 100 μL of the enzymatic extract. PPO activity was expressed in enzyme unit mg^–1^ protein. One unit of PPO activity was defined as the amount of enzyme causing an increase in the absorbance of 0.001/min at 420 nm.

### Malondialdehyde and Hydrogen Peroxide Content

Malondialdehyde (MDA) content in leaves was estimated by homogenizing the frozen leaf powder subamples (0.25 g) in 10 mL of 0.1% (w/v) trichloroacetic acid (TCA) and centrifuging at 18,000 *g* for 10 min as described by Madhava Rao and Sresty (2000). Two milliliters of supernatant were mixed with 2 mL of 20% TCA containing 0.5% Thiobarbituric acid (TBA). The mixture was then heated in a water bath at 100°C for 30 min and immediately cooled in an ice bath. The absorbance was read at 532 nm. The nonspecific turbidity was corrected by subtracting A600 from A532, and the MDA content was calculated as follows: [MDA] = 6.45 (A_532_ − A_600_) − 0.56A_450_.

Hydrogen peroxide (H_2_O_2_) concentration in leaves was determined by the method described by Velikova et al. (2000). Briefly, 0.25 g of the frozen leaf powder were homogenized with 5 mL 10% (w/v) TCA and then centrifuged at 15,000 *x g* for 15 min at 4°C. The supernatant (0.5 mL) was recovered to determine the content of H_2_O_2_ and 0.5 mL of potassium phosphate buffer (10 mM, pH 7) and 1 mL of iodic potassium (1 M) was added. After 1 h of incubation, the absorbance at 390 nm was recorded and plotted against a standard H_2_O_2_ curve. The blank was made by replacing the sample extract by 10% TCA.

### Soil Analyses

At plant harvest, soil physicochemical properties were analyzed on samples taken near the roots. The pH and electrical conductivity (EC) were measured in a diluted soil suspension 1/5 (v/v) using a pH meter HI 9025 and a conductivity meter HI-9033 (Hanna Instruments, Padua, Italy), respectively. Total organic carbon (TOC) and organic matter (OM) were measured according to the method described by Aubert (1978), which consists of the oxidation of organic matter by potassium dichromate in the presence of sulfuric acid. Available P was determined according to Olsen and Sommers method (1982). The amount mineral N available in soil was measured according to the method described by Rodier (1984).

### Statistical Analysis

Data are presented as mean ± SE (standard error) of six independent biological replicates. Data were analyzed by employing one-way analysis of variance (ANOVA) followed by Tukey’s honest significant difference test using a significance level of 5% (*p* ≤ 0.05). Normality of residuals was tested using the Shapiro-Wilk test. Mycorrhizal root colonization rates were arcsin-square root transformed to fit the assumption of normal distribution. Multivariate analysis of variance (MANOVA) was performed using SPSS 10.0 software to determine the interaction among the tested factors (AMF × Bacteria × Compost × Drought). Different lower cases indicate significant differences among treatments at *p* ≤ 0.05. In order to integrate all the data, a complete dataset comprising all growth, physiological, and biochemical data was subjected to Principal Components Analysis (PCA). The PCA was performed using XLSTAT v. 2014.

## Results

### Mycorrhization Parameters

**FIGURE 1:**
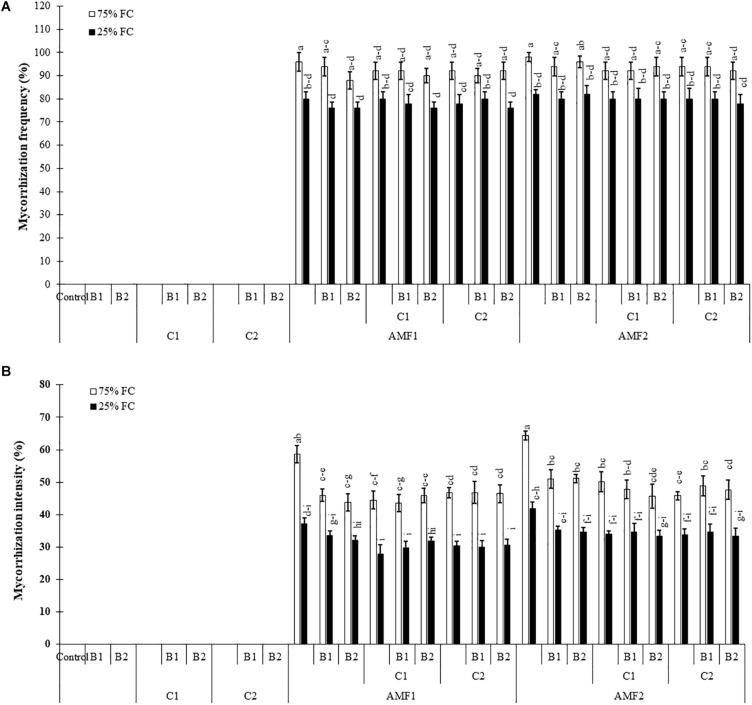
Influence of different water regimes [75% field capacity (FC); open bars and 25% FC; filled bars] on **(A)** mycorrhization frequency and **(B)** intensity in control plants (non-amended, non-inoculated), and plants amended with composts (C1 or C2) and/or inoculated with arbuscular mycorrhizal fungi (AMF, exogenous AMF1 or native AMF2) or plant growth promoting rhizobacteria (PGPR) strains (B1 or B2). Data are mean ± SE of six biological replicates. Means followed by the same letters are not significantly different at *P* < 0.05 (Tukey’s HSD).

Our results showed that no mycorrhizal structure was observed in the roots of non-treatment controls. The frequency and intensity of AMF in date palm roots was significantly decreased by drought stress (Supplementary Table 1). The plants inoculated with AMF, especially for AMF1, without compost and PGPR showed the higher root colonization intensity compared to plants treated with compost and PGPR (Figures 1A,B). AMF infection frequency and intensity showed no significant difference between date palm inoculated with AMF alone or combined with PGPR and/or composts (bi- and tripartite combinations) under drought stress conditions (Figures 1A,B). The interactions between AMF and drought were significant for these two parameters (Supplementary Tables 1A and B).

### Growth Assessment and Mineral Nutrition

**FIGURE 2:**
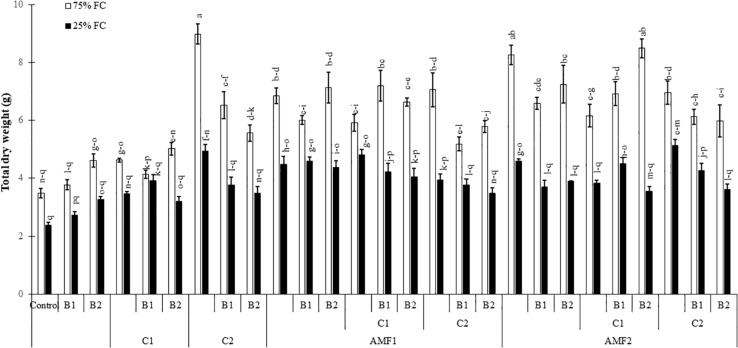
Influence of different water regimes (75% field capacity (FC); open bars and 25% FC; filled bars) on date palm total dry matter in control (non-amended, non-inoculated), and plants amended with composts (C1 or C2) and/or inoculated with arbuscular mycorrhizal fungi (AMF, exogenous AMF1 or native AMF2) or plant growth promoting rhizobacteria (PGPR) strains (B1 or B2) date palms. Data are mean ± SE of six biological replicates. Means followed by the same letters are not significantly different at *P* < 0.05 (Tukey’s HSD).

Drought caused a significant decrease (*P* < 0.001) (Supplementary Table 1) in all the growth parameters such as leaf number, plant height, root length, leaf area (Table 4), and total dry weight (Figure 2). Our results showed that the un-inoculated and un-amended control performed very weak response in all these parameters compared to the treated plants under both well-watered and drought stress conditions (Figure 2, Table 4, and Supplementary Figure 1). Under drought stress, however, the application of bi- and tripartite combinations of biofertilizers (AMF1+C1, AMF1+C1+B1, AMF2+C2+B1, and AMF2+C1+B2) showed positive effects by promoting date palm shoot height and root length to a greater extent than in non-inoculated and non-amended plants. Moreover, the compost alone, bi and tripartite combinations (AMF1+C2+B1, AMF2+C2, C2) increased the number of leaves as compared to non-inoculated and non-amended date palm plants under well-watered and water deficit conditions. The plants treated with AMF1+C1, AMF2+B2, and AMF2+C2 improved the leaf area compared to non-inoculated and non-amended vitroplants under water deficit. A positive effect on the total dry weight of vitroplants subjected to water stress was recorded after application of biofertilizers (Figure 2). Indeed, the AMF and compost alone, bi and tripartite combinations (5 g in AMF2+C2 and C2, 4.8 g in AMF1+C1, and 4.6 g in AMF2, AMF1+B1, and AMF2+C1+B1) showed the highest values of this parameter under water deficit in comparison with non-inoculated and non-amended vitroplants (ca. 2.4 g).

**TABLE 4:** Influence of different water regimes on growth parameters of non-amended and non-inoculated plants (control), and plants amended and inoculated date palm plants with composts (C1 or C2) and/or arbuscular mycorrhizal fungi (AMF, exogenous AMF1 and native AMF2), and/or plant growth promoting rhizobacteria (PGPR) (B1 or B2).

Treatments	Leaf number		Shoot height (cm)		Root length (cm)		Leaf area (cm^2^)	
	75% FC	25% FC	75% FC	25% FC	75% FC	25% FC	75% FC	25% FC
Control	4.6 ± 0.2 fg	3.8 ± 0.2 g	23.6 ± 0.8 lm	21.1 ± 0.6 m	21.9 ± 0.6 n–p	18.5 ± 0.9 p	29.8 ± 1.4 g–j	21.7 ± 0.9 j
B1	5.2 ± 0.2 c–f	4.6 ± 0.2 fg	25.7 ± 0.5 c–m	23.4 ± 0.7 lm	25.2 ± 0.7 d–n	23.2 ± 1.6 i–o	35.0 ± 1.8 c–h	22.7 ± 1.4 ij
B2	6.4 ± 0.2 a–c	5.4 ± 0.2 b–f	27.2 ± 0.4 b–l	23.0 ± 0.7 lm	26.3 ± 1.1 a–m	22.4 ± 1.4 m–p	34.0 ± 1.8 d–h	26.3 ± 1.6 h–j
C1	6.4 ± 0.2 a–c	5.4 ± 0.2 b–f	28.8 ± 1.2 a–j	24.1 ± 0.6 j–m	26.1 ± 0.8 a–m	22.5 ± 1.0 l–p	39.8 ± 1.9 a–f	28.3 ± 1.0 g–j
C1+B1	6.2 ± 0.2 a–d	4.6 ± 0.2 fg	27.5 ± 0.9 b–l	25.0 ± 0.8 f–m	26.7 ± 1.1 a–k	21.9 ± 0.9 n–p	42.5 ± 0.9 a–d	32.7 ± 0.9 f–h
C1+B2	6.0 ± 0.3 a–e	5.0 ± 0.3 d–g	29.8 ± 0.8 a–f	26.6 ± 1.0 b–l	26.6 ± 0.5 a–l	23.4 ± 1.3 i–o	43.3 ± 2.1 a–c	29.3 ± 1.3 g–j
C2	6.8 ± 0.2 a	5.6 ± 0.2 a–f	30.1 ± 0.6 a–d	25.7 ± 0.5 c–m	27.1 ± 0.5 a–j	22.8 ± 0.8 k–o	45.3 ± 1.5 a	34.5 ± 0.8 c–h
C2+B1	6.2 ± 0.2 a–d	5.4 ± 0.2 b–f	27.6 ± 0.9 a–l	25.1 ± 0.9 e–m	24.5 ± 0.2 g–o	21.0 ± 0.4 op	42.0 ± 1.2 a–e	32.0 ± 0.7 f–h
C2+B2	5.8 ± 0.2 a–f	5.4 ± 0.2 b–f	31.0 ± 1.1 ab	26.7 ± 0.9 b–l	28.7 ± 0.9 a–f	23.4 ± 0.5 i–o	48.5 ± 1.7 a	33.0 ± 1.6 f–h
AMF1	6.4 ± 0.2 a–c	5.4 ± 0.2 b–f	30.0 ± 1.3 a–d	27.2 ± 0.5 b–l	27.9 ± 1.3 a–h	23.1 ± 0.4 j–o	43.3 ± 1.7 a–c	31.5 ± 0.7 f–i
AMF1+B1	6.2 ± 0.3 a–d	5.2 ± 0.2 c–f	30.2 ± 0.7 a–c	25.6 ± 0.9 c–m	29.3 ± 0.8 a–d	25.3 ± 0.5 d–n	44.0 ± 1.6 ab	33.0 ± 1.3 f–h
AMF1+B2	6.6 ± 0.2 ab	5.4 ± 0.2 b–f	29.6 ± 0.9 a–g	26.4 ± 0.6 b–l	29.2 ± 1.3 a–d	24.4 ± 0.5 g–o	43.0 ± 1.1 a–c	34.0 ± 1.1 d–h
AMF1+C1	5.6 ± 0.2 a–f	4.8 ± 0.2 e–g	29.9 ± 1.0 a–e	27.6 ± 0.7 a–l	30.0 ± 1.1 a	25.0 ± 0.5 e–o	46.0 ± 1.9 a	34.7 ± 1.5 c–h
AMF1+C1+B1	6.8 ± 0.2 a	5.2 ± 0.2 c–f	30.4 ± 1.3 a–c	27.5 ± 1.1 a–l	29.5 ± 0.5 a–c	27.1 ± 0.5 a–j	45.0 ± 2.2 a	31.3 ± 0.9 f–i
AMF1+C1+B2	5.8 ± 0.2 a–f	5.2 ± 0.2 c–f	29.5 ± 0.5 a–h	26.8 ± 0.9 b–l	30.0 ± 0.8 a	24.9 ± 0.6 e–o	42.0 ± 2.0 a–e	33.0 ± 1.3 f–h
AMF1+C2	6.4 ± 0.2 a–c	5.4 ± 0.2 b–f	28.6 ± 1.2 a–j	24.7 ± 0.7 h–m	28.2 ± 1.2 a–g	26.2 ± 0.6 a–m	45.3 ± 1.7 a	31.7 ± 1.1 f–h
AMF1+C2+B1	5.8 ± 0.2 a–f	5.8 ± 0.2 a–f	27.2 ± 0.8 b–l	24.4 ± 0.7 j–m	27.3 ± 0.4 a–i	24.7 ± 0.5 f–o	44.3 ± 1.9 ab	31.7 ± 1.6 f–h
AMF1+C2+B2	6.2 ± 0.2 a–d	5.0 ± 0.3 d–g	30.2 ± 0.6 a–c	24.8 ± 0.7 g–m	29.0 ± 0.7 a–e	25.6 ± 0.7 b–n	47.8 ± 1.7 a	32.6 ± 1.5 f–h
AMF2	6.2 ± 0.2 a–d	5.4 ± 0.2 b–f	28.9 ± 0.7 a–j	24.8 ± 0.6 g–m	28.8 ± 0.9 a–f	24.4 ± 0.5 g–o	47.8 ± 2.4 a	32.3 ± 1.1 f–h
AMF2+B1	6.0 ± 0.3 a–e	5.2 ± 0.2 c–f	29.5 ± 1.2 a–h	25.9 ± 0.6 c–m	27.9 ± 0.6 a–h	25.2 ± 0.3 d–n	46.3 ± 1.4 a	33.7 ± 1.7 d–h
AMF2+B2	6.2 ± 0.2 a–d	5.4 ± 0.2 b–f	28.5 ± 0.7 a–k	24.6 ± 0.6 i–m	28.8 ± 0.9 a–f	25.4 ± 0.5 c–n	47.5 ± 1.5 a	35.0 ± 0.9 c–h
AMF2+C1	5.8 ± 0.2 a–f	5.0 ± 0.0 d–g	32.3 ± 1.2 a	27.0 ± 0.7 b–l	26.0 ± 0.4 a–n	22.6 ± 0.7 k–p	43.3 ± 1.2 a–c	32.8 ± 1.1 f–h
AMF2+C1+B1	6.2 ± 0.2 a–d	5.4 ± 0.2 b–f	29.4 ± 0.9 a–i	23.8 ± 0.7 k–m	26.1 ± 0.5 a–m	24.0 ± 0.7 h–o	44.5 ± 2.0 ab	34.0 ± 1.4 d–h
AMF2+C1+B2	5.8 ± 0.2 a–f	5.2 ± 0.2 c–f	31.0 ± 0.6 ab	28.4 ± 0.8 a–k	29.6 ± 1.2 ab	27.1 ± 0.7 a–j	45.3 ± 2.2 a	33.5 ± 1.6 e–h
AMF2+C2	6.2 ± 0.2 a–d	5.6 ± 0.2 a–f	29.9 ± 1.3 a–e	26.8 ± 0.4 b–l	26.2 ± 0.8 a–m	23.5 ± 0.5 i–o	46.0 ± 2.1 a	36.0 ± 1.1 b–g
AMF2+C2+B1	6.0 ± 0.3 a–e	4.8 ± 0.2 e–g	32.3 ± 1.1 a	27.3 ± 0.9 b–l	28.5 ± 0.4 a–g	24.8 ± 0.6 f–o	45.0 ± 1.9 a	32.3 ± 1.4 f–h
AMF2+C2+B2	6.0 ± 0.0 a–e	4.8 ± 0.0 e–g	29.9 ± 0.6 a–d	25.3 ± 0.5 d–m	26.5 ± 0.6 a–m	22.7 ± 0.7 k–o	44.5 ± 0.8 ab	33.8 ± 1.6 d–h

*Means followed by the same letters are not significantly different *P* < 0.05 (Tukey’s HSD).*

**FIGURE 3:**
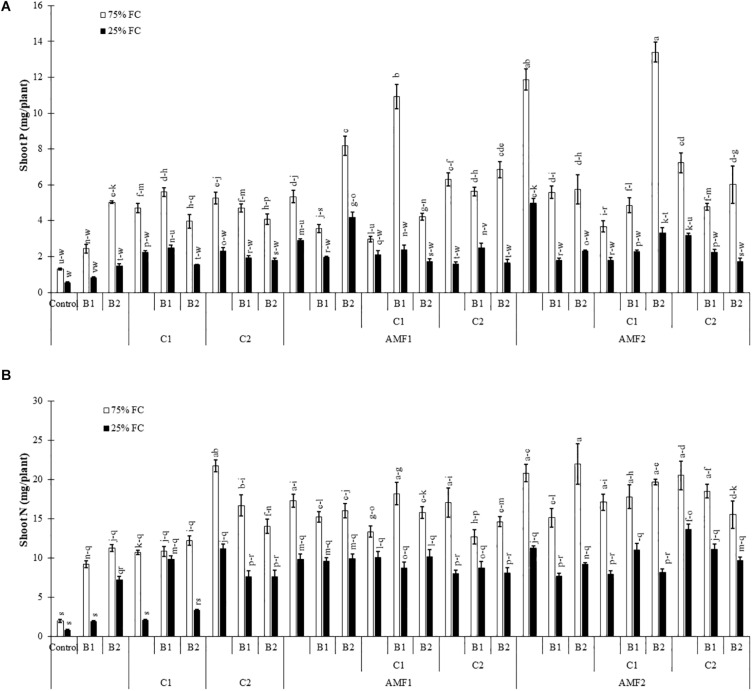
**(A)** Phosphorous (P) and **(B)** nitrogen (N) content in date palm shoots under two water regimes [75% field capacity (FC); open bars and 25% FC; filled bars] of the tested control (non-amended and non-inoculated) and biofertilizers treatments [composts C1 or C2, arbuscular mycorrhizal fungi (AMF, exogenous AMF1 and native AMF2), and/or plant growth promoting rhizobacteria (PGPR) (B1 or B2)]. Data are mean ± SE of six biological replicates. Means followed by the same letters are not significantly different at *P* < 0.05 (Tukey’s HSD).

We assayed the P and N content in shoots of date palm plants under drought and different biofertilizers, since the degree of stress and growth depend on their uptake and translocation. Under the control condition (75% FC), shoot P was significantly increased in plants treated with AMF (Figure 3A) as compared to non-amended and non-inoculated control plants, whereas under drought stress, it was decreased. Under water deficit, shoot P content was significantly increased by C1+B1, AMF1, AMF1+B2, AMF1+C1+B1, AMF2, AMF1+B1+C1AMF2+C1+B2, and AMF2+C2 in comparison with non-treated plants (Figure 3A). Under 75% FC, N levels in leaves of all treated plants remained significantly higher than in control conditions. Drought stress decreased N content in all treatments, and all the biofertilizer treatments were able to maintain higher content than non-amended and non-inoculated control plants (Figure 3B). The interaction between drought × C1 × B1 (Supplementary Table 1A), drought × B2, and drought × C1 × B2 (Supplementary Table 1B) had a significant effect (*P* < 0.01) on P, while N showed a significant effect between drought × AMF2 (Supplementary Table 1B).

### Physiological Traits

**FIGURE 4:**
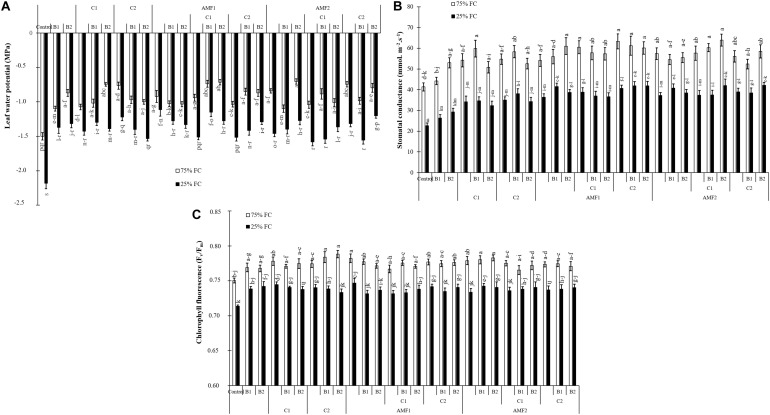
**(A)** Leaf water potential, **(B)** stomatal conductance, and **(C)** chlorophyll fluorescence of date palm plants under two water regimes [75% field capacity (FC); open bars and 25% FC; filled bars] and grown under control (non-amended and non-inoculated) or biofertilizer applications [composts C1 or C2, arbuscular mycorrhizal fungi (exogenous AMF1 and native AMF2), and/or plant growth promoting rhizobacteria (PGPR) (B1 or B2)]. Data are mean ± SD of six biological replicates. Means followed by the same letters are not significantly different at *P* < 0.05 (Tukey’s HSD).

All physiological parameters were significantly decreased by drought (*P* < 0.001) (Supplementary Table 1). Under water scarcity, the leaf water potential values were decreased in non-inoculated and non-amended control plants. Plant inoculation with AMF and/or PGPR amended or not with the compost yielded an improvement in leaf water potential under water deficit, especially AMF1 (−1 MPa), AMF1+C1+B1 (−1.15 MPa), and AMF2+C2+B2 (−1.20 MPa) versus non-inoculated and no-amended plants (−2.18 MPa) (Figure 4A).

Under water control condition, there was obvious stomatal conductance differences between non-amended/non-inoculated and treated plants with AMF and/or PGPR. Under water stress, stomatal conductance values decreased in date palm plants. However, the application of biofertilizers increased stomatal conductance, with AMF1 alone, the bi- (C2+B1, AMF1+B1, AMF1+B2, AMF1+C2, and AMF2+B1) and tripartite (AMF1+C2+B1, AMF1+C2+B2, AMF2+C1+B2, and AMF2+C2+B2) combinations being the most effective in improving this parameter compared to control plants (Figure 4B).

As shown in Figure 4C, the chlorophyll fluorescence (F_v_/F_m_) was only slightly affected by drought stress. Biofertilizer application improved F_v_/F_m_ in date palm plants under water shortage. The single (AMF1, PGPR B2 and compost C1), bi- (AMF1+C2), and tripartite (AMF2+C1+B2) combinations presented the most effective treatments to increase chlorophyll fluorescence under water deficit conditions compared to non-inoculated and non-amended plants.

**FIGURE 5:**
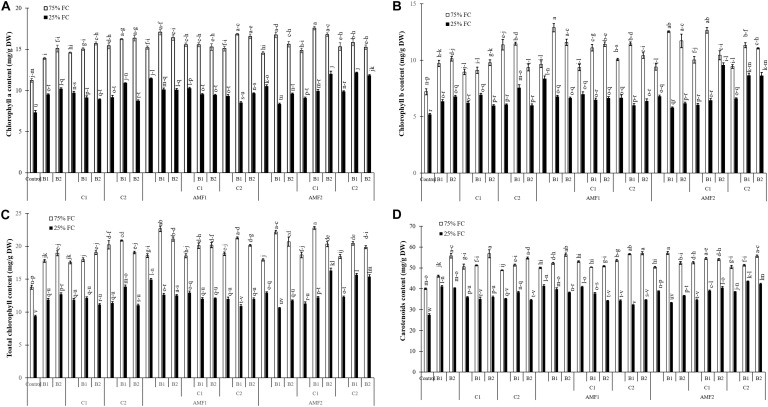
**(A)** Chlorophyll *a*, **(B)** chlorophyll *b*, **(C)** total chlorophyll, and **(D)** carotenoid content in leaves of date palm plants under two water regimes [75% field capacity (FC); open bars and 25% FC; filled bars] and further grown without (control; non-amended and non-inoculated) or with biofertilizers [composts C1 or C2, arbuscular mycorrhizal fungi (AMF, exogenous AMF1 and native AMF2), and/or PGPR (B1 or B2)]. Data are mean ± SE of six independent biological replicates. Means followed by the same letters are not significantly different at *P* < 0.05 (Tukey’s HSD).

In response to drought stress and inoculation with AMF, PGPR and compost application, chlorophyll *a*, *b*, total chlorophyll, and carotenoid content are shown in Figure 5. Under water deficit, the photosynthetic pigment content was reduced. However, the application of AMF, compost, and PGPR especially the combination C2+B1, AMF2+C1+B2, AMF2+C2+B1, AMF1, and AMF2+C2+B2 increased pigments contents compared to control plants, under water stress conditions. As for carotenoid content, this was positively affected by biofertilizers applied alone (B1, B2, C2, C1, AMF2, and AMF1) or in combination (AMF2+C1, AMF2+C1+B1, AMF1+B2, AMF2+C2+B2, AMF2+C1+B2, and AMF2+C2+B1) as compared with non-inoculated with AMF/PGPR and non-amended with composts, under water deficit.

### Biochemical Traits

**FIGURE 6:**
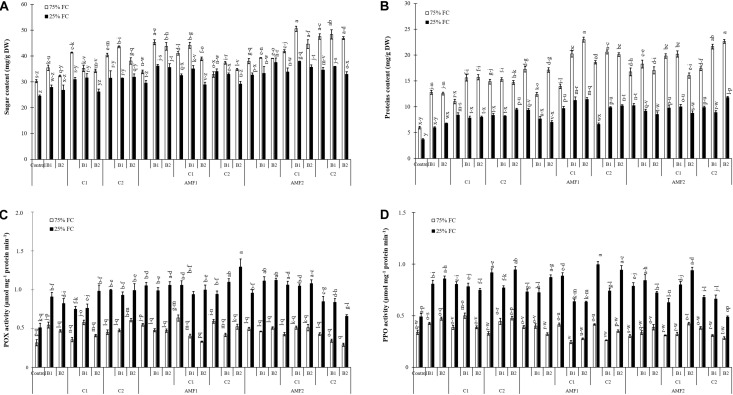
**(A)** Total soluble sugar content, **(B)** protein content, **(C)** peroxidase (POX) activity, and **(D)** polyphenol oxidase (PPO) activity in date palm shoots under two water regimes [75% field capacity (FC); open bars and 25% FC; filled bars] of the tested control treatments (non-amended and non-inoculated) and biofertilizers treatments [composts C1 or C2, arbuscular mycorrhizal fungi (exogenous AMF1 and native AMF2), and/or plant growth promoting rhizobacteria (PGPR) (B1 or B2)]. Data are mean ± SE of six biological replicates. Means followed by the same letters are not significantly different at *P* < 0.05 (Tukey’s HSD).

Treatment effects on biochemical traits were significantly decreased by drought (*P* < 0.001) (Supplementary Table 1). Results related to the effect of drought stress and biofertilizer applications on sugar and protein content and POX and PPO activities in date palm plants are presented in Figure 6. Under normal water conditions, both compost and AMF increased sugar and protein content. Exposure to water deficit caused a significant decrease in sugar and protein content (Figures 6A,B). The addition of biofertilizers yielded a significant increase in sugar and protein compared to stressed control plants. Under 75% FC conditions, POX and PPO did not differ significantly among the biofertilizers treatments (Figures 6C,D) Exposure to drought stress led to a considerable increase in the POX and PPO specific activities as compared to non-treated control plants.

**FIGURE 7:**
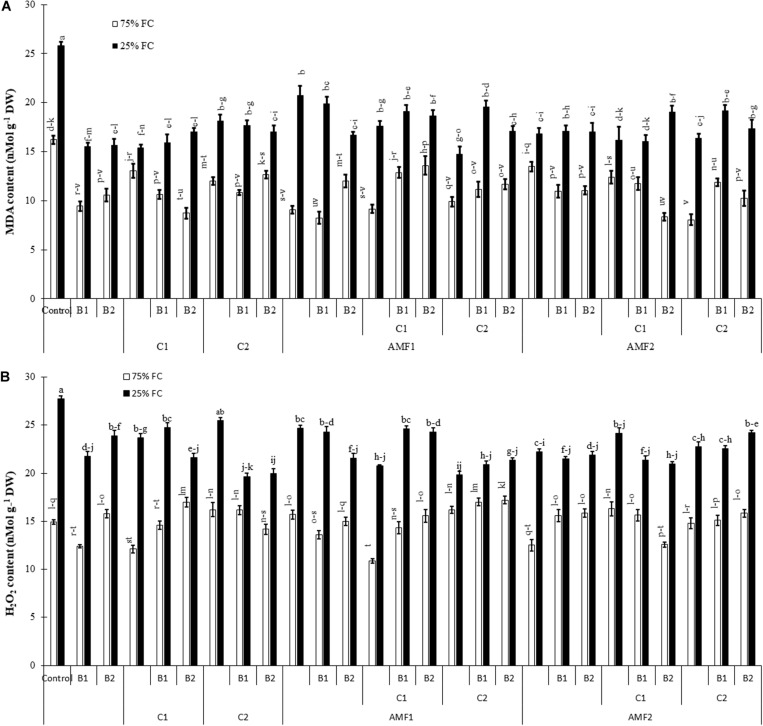
**(A)** Malondialdehyde (MDA) and **(B)** hydrogen peroxide (H_2_O_2_) content in date palm shoots under two water regimes (75% field capacity (FC); open bars and 25% FC; filled bars) of the tested control treatments (non-amended and non-inoculated) and biofertilizers treatments [composts C1 or C2, arbuscular mycorrhizal fungi (AMF, exogenous AMF1 and native AMF2), and/or plant growth promoting rhizobacteria (PGPR) (B1 or B2)]. Data are mean ± SE of six independent biological replicates. Means followed by the same letters are not significantly different at P < 0.05 (Tukey’s HSD).

To characterize damage caused by drought stress, we carried out MDA and H_2_O_2_ analyses (Figure 7). The exposure of date palm plants to severe water deficit resulted in an increase in MDA and H_2_O_2_ content. Under water stress, in contrast, the application of single or combined biofertilizers showed reduced MDA and H_2_O_2_ content compared to non-inoculated and non-amended controls. The interactions AMF1 × C2 × Drought, and AMF1 × B1 × Drought (Supplementary Table 1A), AMF2 × C2 × Drought, B1 × Drought, and B1 × Drought (Supplementary Table 1B) had a significant effect on H_2_O_2_ content.

### Soil Analysis

We assayed the pH, electrical conductivity, total organic carbon, total organic matter, N, and P content in the soil just after harvesting the plants, since the degree of soil quality depends on their values after the culture (Table 5). Under control conditions, the addition of composts increased the soil quality more than controls. Under drought stress, soil analyses at harvest time showed that biofertilizers application - mainly composts - improved soil quality as compared to the controls (Table 5). As a result, relative to control plants, the soil pH was decreased following the application of biofertilizers after prolonged drought. Under these conditions, EC, TOC, and OM were improved by biofertilizers. Moreover, under drought stress, both total N and P content in the soil were improved when biofertilizers were applied, especially in the treatments AMF1+B2, AMF1+C2+B1, AMF2, AMF2+B2, AMF2+C1+B2, and AMF2+C2.

**TABLE 5:** Soil physic-chemical analysis at harvest time of date palm grown under two water regimes (75 and 25% Field Capacity (FC)) of the tested control (non-amended and no-inoculated) and biofertilizers (composts C1 or C2, arbuscular mycorrhizal fungi (AMF, exogenous AMF1 and native AMF2), and/or plant growth promoting rhizobacteria (PGPR) (B1 or B2). Data are mean ± SE of six biological replicates.

Treatments	pH		EC (mS/cm)		TOC (%)		OM (%)		N (mg/g of soil)		P (ppm)	
	75% FC	25% FC	75% FC	25% FC	75% FC	25% FC	75% FC	25% FC	75% FC	25% FC	75% FC	25% FC
Control	7.96 ± 0.05 a	7.88 ± 0.06 ab	0.28 ± 0.01 v–x	0.20 ± 0.01 A	0.40 ± 0.02 v	0.52 ± 0.01 u	0.70 ± 0.03 v	0.89 ± 0.02 u	0.16 ± 0.02 lm	0.06 ± 0.02 m	51.97 ± 1.47 u–w	30.05 ± 1.44 w
B1	7.50 ± 0.07 c–e	7.46 ± 0.02 c–e	0.23 ± 0.00 y–A	0.21 ± 0.01 zA	0.75 ± 0.01 n–s	0.71 ± 0.01 p–t	1.29 ± 0.02 n–s	1.22 ± 0.02 p–t	0.30 ± 0.03 e–k	0.22 ± 0.02 j–l	86.53 ± 5.37 m–t	41.34 ± 1.64 vw
B2	7.38 ± 0.04 c	7.42 ± 0.02 de	0.25 ± 0.01 x–z	0.21 ± 0.01 zA	0.68 ± 0.06 st	0.74 ± 0.02 o–s	1.16 ± 0.10 st	1.28 ± 0.04 o–s	0.30 ± 0.03 e–k	0.22 ± 0.02 j–l	151.48 ± 7.02 c–e	57.97 ± 3.46 s–w
C1	7.46 ± 0.05 c–e	7.56 ± 0.02 c–e	0.30 ± 0.01 s–w	0.31 ± 0.01 r–v	0.87 ± 0.03 e–l	0.83 ± 0.01 h–o	1.51 ± 0.05 e–l	1.43 ± 0.03 h–o	0.24 ± 0.02 j–l	0.34 ± 0.04 b–k	140.04 ± 11.28 c–g	86.59 ± 3.88 m–s
C1+B1	7.44 ± 0.05 c–e	7.54 ± 0.02 c–e	0.39 ± 0.01 h–n	0.39 ± 0.01 h–n	0.94 ± 0.02 b–g	0.90 ± 0.00 e–k	1.62 ± 0.03 b–g	1.55 ± 0.01 e–k	0.34 ± 0.04 b–k	0.22 ± 0.02 j–l	190.18 ± 9.77 b	80.89 ± 1.68 n–u
C1+B2	7.52 ± 0.04 c–e	7.48 ± 0.04 c–e	0.38 ± 0.01 i–o	0.21 ± 0.01 zA	0.92 ± 0.01 d–i	0.84 ± 0.01 g–n	1.58 ± 0.03 d–i	1.46 ± 0.02 g–n	0.32 ± 0.02 c–k	0.25 ± 0.01 j–l	105.63 ± 8.57 h–n	64.06 ± 1.36 q–v
C2	7.42 ± 0.04 de	7.48 ± 0.04 c–e	0.38 ± 0.01 h–o	0.37 ± 0.01 j–p	0.94 ± 0.02 b–g	0.87 ± 0.02 e–l	1.62 ± 0.03 b–g	1.50 ± 0.03 e–l	0.47 ± 0.02 ab	0.28 ± 0.02 g–l	79.43 ± 3.91 n–u	61.90 ± 2.57 r–v
C2+B1	7.52 ± 0.05 c–e	7.58 ± 0.02 c–e	0.32 ± 0.00 q–v	0.31 ± 0.00 r–v	0.95 ± 0.02 b–f	0.84 ± 0.02 g–o	1.64 ± 0.03 b–f	1.44 ± 0.03 g–o	0.35 ± 0.02 a–k	0.21 ± 0.02 kl	96.76 ± 3.17 k–p	71.07 ± 3.07 o–u
C2+B2	7.50 ± 0.04 c–e	7.58 ± 0.04 c–e	0.35 ± 0.01 n–r	0.33 ± 0.01 p–u	0.91 ± 0.02 e–j	0.68 ± 0.01 r–t	1.57 ± 0.03 e–j	1.16 ± 0.03 r–t	0.32 ± 0.02 c–k	0.26 ± 0.02 i–l	100.34 ± 2.42 j–o	69.50 ± 4.30 p–v
AMF1	7.56 ± 0.02 c–e	7.52 ± 0.04 c–e	0.25 ± 0.01 x–z	0.23 ± 0.00 y–A	0.75 ± 0.01 n–s	0.68 ± 0.01 r–t	1.29 ± 0.02 n–s	1.17 ± 0.03 r–t	0.29 ± 0.02 f–l	0.32 ± 0.02 c–k	114.85 ± 5.74 g–m	87.29 ± 4.24 m–s
AMF1+B1	7.64 ± 0.02 b–d	7.56 ± 0.02 c–e	0.25 ± 0.01 x–z	0.27 ± 0.01 w–y	0.70 ± 0.03 p–t	0.78 ± 0.00 l–q	1.21 ± 0.05 p–t	1.35 ± 0.01 l–q	0.39 ± 0.03 a–i	0.34 ± 0.02 b–k	80.54 ± 2.33 n–u	57.87 ± 2.25 s–w
AMF1+B2	7.68 ± 0.04 bc	7.56 ± 0.02 c–e	0.30 ± 0.01 t–w	0.29 ± 0.01 u–x	0.70 ± 0.01 q–t	0.64 ± 0.02 t	1.20 ± 0.01 q–t	1.10 ± 0.04 t	0.32 ± 0.02 c–k	0.28 ± 0.02 g–l	156.47 ± 8.83 cd	126.84 ± 1.00 e–j
AMF1+C1	7.48 ± 0.04 c–e	7.54 ± 0.02 c–e	0.39 ± 0.01 h–o	0.31 ± 0.01 r–w	0.90 ± 0.00 e–j	0.80 ± 0.01 k–p	1.56 ± 0.00 e–j	1.38 ± 0.02 k–p	0.41 ± 0.03 a–g	0.31 ± 0.01 d–k	68.55 ± 1.05 p–v	58.47 ± 3.04 r–w
AMF1+C1+B1	7.50 ± 0.09 c–e	7.48 ± 0.04 c–e	0.45 ± 0.01 d–g	0.34 ± 0.01 o–t	0.97 ± 0.01 a–e	0.77 ± 0.02 m–r	1.67 ± 0.01 a–e	1.32 ± 0.03 m–r	0.31 ± 0.03 d–k	0.25 ± 0.01 j–l	207.15 ± 3.30 ab	74.39 ± 3.45 o–u
AMF1+C1+B2	7.64 ± 0.02 b–d	7.68 ± 0.04 bc	0.41 ± 0.01 g–k	0.32 ± 0.01 q–v	1.07 ± 0.00 a	0.89 ± 0.02 e–k	1.85 ± 0.00 a	1.54 ± 0.04 e–k	0.36 ± 0.03 a–j	0.31 ± 0.01 d–k	86.99 ± 1.47 m–s	57.06 ± 2.51 t–w
AMF1+C2	7.65 ± 0.05 c–e	7.68 ± 0.04 bc	0.49 ± 0.01 cd	0.41 ± 0.01 f–j	0.90 ± 0.01 e–k	1.03 ± 0.01 a–c	1.55 ± 0.02 e–k	1.78 ± 0.01 a–c	0.44 ± 0.02 a–e	0.31 ± 0.02 d–k	127.90 ± 12.23 d–j	58.22 ± 3.26 s–w
AMF1+C2+B1	7.66 ± 0.06 b–d	7.68 ± 0.04 bc	0.55 ± 0.01 b	0.42 ± 0.01 e–i	1.05 ± 0.02 ab	0.93 ± 0.00 c–h	1.80 ± 0.03 ab	1.60 ± 0.01 c–h	0.32 ± 0.02 c–k	0.29 ± 0.02 f–l	149.41 ± 8.46 c–f	87.94 ± 3.18 m–r
AMF1+C2+B2	7.48 ± 0.07 c–e	7.68 ± 0.04 bc	0.45 ± 0.00 d–f	0.40 ± 0.01 h–m	1.02 ± 0.01 a–d	0.82 ± 0.01 i–o	1.77 ± 0.02 a–d	1.41 ± 0.02 i–o	0.31 ± 0.02 a–d	0.29 ± 0.02 f–l	157.48 ± 7.11 c	66.08 ± 3.09 q–v
AMF2	7.60 ± 0.03 c–e	7.66 ± 0.04 b–d	0.35 ± 0.01 m–r	0.32 ± 0.01 q–v	0.53 ± 0.00 u	0.64 ± 0.01 t	0.91 ± 0.00 u	1.10 ± 0.02 t	0.35 ± 0.03 a–k	0.34 ± 0.03 b–k	198.64 ± 8.48 ab	149.41 ± 8.35 c–f
AMF2+B1	7.56 ± 0.05 c–e	7.68 ± 0.04 bc	0.33 ± 0.01 p–u	0.28 ± 0.01 v–x	0.66 ± 0.01 st	0.78 ± 0.01 l–q	1.14 ± 0.02 st	1.35 ± 0.01 l–q	0.45 ± 0.03 a–d	0.31 ± 0 01 d–k	121.65 ± 2.58 f–k	65.12 ± 2.55 q–v
AMF2+B2	7.66 ± 0.04 b–d	7.60 ± 0.03 c–e	0.37 ± 0.01 k–p	0.36 ± 0.01 l–q	0.85 ± 0.02 f–m	0.84 ± 0.01 g–o	1.47 ± 0.04 f–m	1.44 ± 0.03 g–o	0.35 ± 0.03 a–k	0.36 ± 0.03 a–j	108.20 ± 5.69 h–n	83.21 ± 0.96 n–t
AMF2+C1	7.58 ± 0.04 c–e	7.64 ± 0.02 b–d	0.43 ± 0.01 e–h	0.38 ± 0.01 i–o	1.05 ± 0.01 ab	0.75 ± 0.01 n–s	1.80 ± 0.02 ab	1.30 ± 0.02 n–s	0.45 ± 0.02 a–d	0.29 ± 0.02 f–l	82.05 ± 1.52 n–t	62.10 ± 3.00 r–v
AMF2+C1+B1	7.64 ± 0.05 b–d	7.56 ± 0.04 c–e	0.42 ± 0.01 e–i	0.34 ± 0.01 n–s	0.92 ± 0.02 d–i	0.69 ± 0.01 q–t	1.59 ± 0.03 d–i	1.19 ± 0.01 q–t	0.40 ± 0.03 a–h	0.32 ± 0.02 c–k	92.03 ± 2.27 l–q	64.32 ± 1.73 q–v
AMF2+C1+B2	7.66 ± 0.04 b–d	7.52 ± 0.04 c–e	0.48 ± 0.01 cd	0.40 ± 0.01 h–l	0.81 ± 0.01 i–o	0.92 ± 0.02 d–i	1.40 ± 0.01 i–o	1.58 ± 0.04 d–i	0.48 ± 0.02 a	0.32 ± 0.02 c–k	223.33 ± 3.07 a	121.35 ± 6.35 f–l
AMF2+C2	7.54 ± 0.04 c–e	7.58 ± 0.05 c–e	0.65 ± 0.01 a	0.51 ± 0.01 bc	0.85 ± 0.02 f–m	0.90 ± 0.01 e–j	1.47 ± 0.03 f–m	1.55 ± 0.02 e–j	0.43 ± 0.03 a–f	0.32 ± 0.04 c–k	134.55 ± 6.93 c–h	81.90 ± 3.92 n–t
AMF2+C2+B1	7.64 ± 0.02 b–d	7.52 ± 0.04 c–e	0.51 ± 0.01 bc	0.42 ± 0.01 e–i	0.83 ± 0.01 h–o	0.84 ± 0.01 g–n	1.42 ± 0.02 h–o	1.45 ± 0.03 g–n	0.46 ± 0.02 a–c	0.27 ± 0.02 h–l	104.17 ± 3.09 i–n	70.97 ± 4.55 o–u
AMF2+C2+B2	7.68 ± 0.06 bc	7.54 ± 0.04 c–e	0.62 ± 0.01 a	0.46 ± 0.01 de	1.07 ± 0.00 a	0.90 ± 0.02 e–k	1.84 ± 0.01 a	1.55 ± 0.03 e–k	0.36 ± 0.03 a–j	0.26 ± 0.01 i–l	132.43 ± 9.97 c–i	61.90 ± 2.82 r–v

*Means followed by the same letters are not significantly different at *P* < 0.05 (Tukey’s HSD). EC, electrical conductivity; TOC, total organic carbon; OM, organic matter, N, nitrogen concentration; P, phosphorous concentration.*

### Principal Component Analysis (PCA)

**FIGURE 8:**
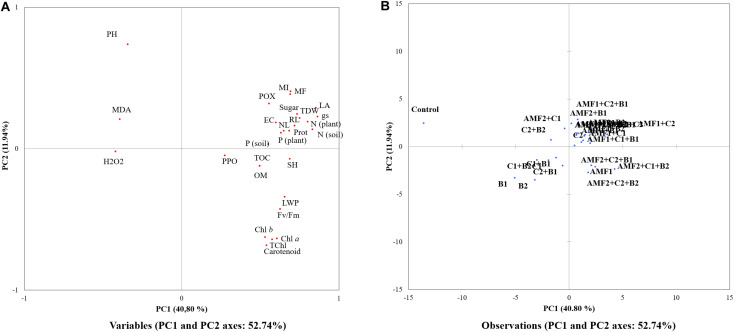
Principal component analysis (PCA) of the different studied **(A)** traits and **(B)** treatments under drought stress conditions (25% FC). Chl a, chlorophyll *a*; Chl b, chlorophyll *b*; EC, electrical conductivity; Fv/Fm, chlorophyll fluorescence; g_s_, stomatal conductance; H_2_O_2_, hydrogen peroxide; LA, leaf area; LWP, leaf water potential; MDA, malondialdehyde; MI, mycorrhizal intensity; MF, mycorrhizal frequency; N (soil), nitrogen content in soil; N (plant); nitrogen content in plant; NL, leaf numbers; OM, organic matter; P (soil), Phosphorous content in soil; P (plant), Phosphorous content in plant shoot; POX, peroxidase; PPO, polyphenol oxidase; RL, root length; SH, shoot height; TOC, total organic carbon; T Chl, total chlorophylls.

The PCA showed that AMF alone or combined with compost and/or PGPR were the most effective treatments to improve growth, nutrition, osmolytes and antioxidant traits (shown in the right panel of Figure 8A) under drought stress (Figure 8A). PC1 explained 40.8% and PC2 explained 11.9% of the total variance. Figure 8B showed that all biofertilizer treatments, single or combined (right panel of Figure 8B), were separate from the control. In Figure 8A, we observed in the right lower panel of the PC1 component, that the traits PPO, F_v_/F_m_, OM, TOC, Total Chlorophyll, chlorophyll *a* and *b*, shoot height, carotenoid, and leaf water potential were closely related to soil P and N concentration, shoot P and N content, sugar, root length, leaf area, leaf number, POX, protein, mycorrhizal frequency and intensity, and EC. In contrast, relative to biofertilizer treatments, the non-treatment control separated in the left of the PC1 component (Figure 8B) and was related to H_2_O_2_, MDA and pH traits (Figure 8A).

## Discussion

In the present study, the application of composts together with inoculation of the exotic and native AMF and PGPR strains inoculations either as single or combined treatments were very effective in helping date palm plants to attenuate the detrimental effects of drought stress on growth, photosynthetic apparatus, nutrient uptake, physiological traits, and oxidative stress. The treated biofertilizers assayed showed a high level of osmotic stress tolerance under water deficit. Our results show that no mycorrhizal structure was observed in the roots of non-treatment controls, but the plants inoculated with AMF were successfully infected by the native or exogenous mycorrhizal consortium under both well-watered and drought conditions. In the presence of water stress, mycorrhization intensity decreased in the AMF treatments alone or in combination with compost and/or PGPR. Our results are in line with several studies showing that mycorrhizal infection decreased when the host plants were exposed to drought stress (Baslam and Goicoechea, 2012; Meddich et al., 2015a; Paymaneh et al., 2019). Under drought stress, however, our findings show an increase of the mycorrhization intensity in date palms treated by compost and PGPR alone or in combination. Cavagnaro (2014) and Kohler et al. (2015) reported that the application of compost at a low dose (2 and 12.5%) increased AMF infection in the root system. Other studies have shown a positive effect of PGPR on enhancing root infection by AMF (Ben-Laouane et al., 2019; Raklami et al., 2019). On the other hand, Sghir et al. (2014) noted that mycorrhizal frequencies and arbuscular content decreased significantly in palm trees inoculated with the combination AMF-PGPR *Trichoderma harzianum* as compared to plants inoculated only with AMF because it colonizes the same space as AMF. However, dual-inoculation AMF-Trichoderma led to the best date palm growth (Sghir et al., 2014). The application of different biofertilizers (alone or in combination) benefited plant growth (mainly leaf number, shoot height, root length, leaf area, and total dry biomass production). The beneficial effect of mycorrhizal and PGPR associations and compost amendment on growth of date palms, under water deficit, could be explained by the greater uptake of nutrients with low mobility such as P and N contained in the substrate. Previous studies demonstrated that date palm and other plants inoculated with AMF accumulated more N and P in leaves than non-mycorrhizal plants when subjected to drought stress (Meddich et al., 2015c; Hao et al., 2019). Javan Gholiloo et al. (2019) showed that the application of biofertilizers (AMF and PGPR) improve the P and N nutrition and consequently enhances date palm plant growth under deficit conditions. Nadeem and colleagues (2014) showed that AMF and PGPR can regulate mineral nutrition by solubilizing nutrients in the soil and producing plant growth regulators (i.e., hormones). Several studies have indicated that compost, AMF and PGPR improve plant growth through the assimilation of immobile soil nutrients such as N and P (Yadav et al., 2013; Baslam et al., 2014; Al-Karaki, 2016; Barje et al., 2016; Frosi et al., 2016; Yang et al., 2018; Raklami et al., 2019; Yu et al., 2019). Here we showed that inoculated and amended date palm plants had considerably higher mineral nutrient content (P and N) as compared to controls under water deficit conditions allowing higher plant performance. This resulted from the better absorption of the surface area provided by extensive fungal hyphae (Wu and Zou, 2017; Zhang et al., 2018) and/or a direct uptake from compost to plant root (Kohler et al., 2015) and/or the mobilization and absorption of various nutrients from soil to plants by PGPR (Grobelak et al., 2015). AMF-colonization results in the establishment of extensive hyphal networks and glomalin secretion, fueling plants with water and nutrient, and thereby, enhancing soil structure (Pagano, 2014). Recently, Volpe et al. (2016) reported the role of PT family genes as components of the Pi-sensing machinery in root tips, which is up-regulated in AMF colonized plants. In addition to P and N, evidence of the role of AMF and PGPR symbiosis in the “transportome” of several mineral nutrients has been obtained in studies on several plant species (Hogekamp et al., 2011; Casieri et al., 2013).

Physiological traits constitute an important tool to study the effect of drought stress on many plants. Our results showed an increase in leaf water potential, stomatal conductance, F_v_/F_m_, and chlorophyll pigment synthesis in plants inoculated with AMF and PGPR and/or amended by composts. This improvement of physiological traits can lead to an increase in CO_2_ assimilation for photosynthesis. Similarly, several studies have demonstrated the capacity of AMF inoculation to reduce the negative effect of drought stress on date palm growth by improving photosynthesis, water status, and antioxidant activity (Baslam et al., 2014; Meddich et al., 2015a). In our research under drought stress, treated plants reduced the degradation of total chlorophyll *a* and *b* and carotenoid. A higher photosynthetic pigment under drought stress conditions suggests a better performance of the photosynthetic apparatus. Our data show that biofertilizers not only increase water and nutrient uptake to mitigate the negative effect of drought but also improve stomatal conductance. Several studies have reported the existence of a positive correlation between photosynthetic efficiency maintenance and tolerance to drought stress in plants amended with compost and/or inoculated with AMF/PGPR (Wu et al., 2006; Sandhya et al., 2010; Tartoura, 2010; Abd El-Mageed et al., 2018, 2019; Duo et al., 2018; Khosravi Shakib et al., 2019). In response to drought stress, plants treated with biofertilizers showed lower levels of potentials and higher water content, allowing the inoculated and amended plants to sustain high organ hydration and turgor level which maintain overall physiological activities of the cells, especially those linked to the photosynthetic apparatus. Another study showed that the positive effect of compost on soil was related to the improvement of water retention (Tartoura, 2010). The association with AMF amends the plants’ water regulation by triggering hormonal signaling such as ABA-mediating stomatal conductance or by stimulating osmolytes. Other studies showed, under drought stress, the development of microorganisms-mediated mechanisms including modifications in the content of plant hormones (e.g., strigolactones, jasmonic acid, and abscisic acid) and improvement in plant water status by increasing hydraulic conductivity (Chaumont and Tyerman, 2014; Fernández-Lizarazo and Moreno-Fonseca, 2016). The increase in root hydraulic conductivity can be related to an enhanced expression in fungal or plant AQPs (Sánchez-Romera et al., 2016). Chitarra et al. (2016) showed an enhancement in the water transport capacity of treated roots, which correlated with overexpression of the NIP AQP-encoding gene (LeNIP3;1). Recently, Xie et al. (2018) found up-regulation of the root AQP gene PIP expression under moderate water deficit in AMF roots.

Soil organic matter and total organic carbon were improved by the biofertilizers used. This improvement could be explained by a direct contribution from compost or by the ability of AMF and PGPR to metabolize different compounds produced by plant roots mainly carbohydrates and organic acids. Shen et al. (2013) and Ning et al. (2017) showed that inoculation with microorganisms and compost application to plants was very effective in improving soil quality especially in organic matter, water retention, and mineral nutrition. The results obtained showed that biological treatments had an important effect on N and P in soil. However, a high amount of N and P in the rhizosphere soil of plants amended with compost and inoculated with AMF and PGPR could be due to a direct absorption via compost or by the fact that AMF and PGPR efficiently and directly take up from the soil to the plant nutrients such as N and P (Grobelak et al., 2015; Liu et al., 2018). In order to tolerate drought stress, plant adaptation is associated with high concentration of solutes such as soluble sugars and protein to regulate the osmotic potential of cells which, in turn, induce an improvement in water absorption under unfavorable condition (Zhang et al., 2010; Liao et al., 2019). Our data indicated that the concentrations of soluble sugars and protein in leaves increased during drought stress in treated plants as compared to microbes-free controls. These results are in agreement with previous reports using AMF or PGPR (Abbaspour et al., 2012; Vurukonda et al., 2016; Javan Gholiloo et al., 2019). In fact, PGPR were shown to secrete osmolytes to mitigate drought stress, which act synergistically with plants internal osmolytes boosting plant growth (Paul et al., 2008). Bano et al. (2013) and Sandhya et al. (2010) reported that plants inoculated with PGPR and grown under drought stress improved their growth by soluble sugars and protein accumulations compared to non-treated plants. However, our results suggest that the application of compost and inoculation with AMF and PGPR were propitious to carbohydrate accumulation, mainly soluble sugars, in drastic conditions resulting in reduced osmotic potentials in host cells. Indeed, the osmotic stress induced by drought is tolerated by the host plant by altering biochemical responses via the enhancement of metabolite biosynthesis (e.g., sugars and proline) that function as osmolytes, and thereby maintaining the water potential, hydration and turgor level which maintain overall physiological activities under harsh environments. Previous studies showed that sugar metabolism-related genes tend to be enriched in plants treated with beneficial microbes under drought stress (Ahanger et al., 2014; Bárzana et al., 2015). Our results demonstrated that levels of MDA and H_2_O_2_ in leaves were lower under drought stress in treated plants compared to beneficial microbe-free control plants. To explain the low lipid peroxidation damage in AMF-treated plants, two possibilities were suggested by Abbaspour et al. (2012): (1) either inoculated plants with AMF suffered less drought stress owing to a primary drought avoidance effect by symbiosis (e.g., direct water uptake by fungal hyphae from the soil to the host plant) or (2) AMF colonization improved the activities of antioxidant enzymes as a defense to eliminate the ROS. Our results suggest that the application of compost and inoculation with AMF and PGPR could improve the defense against drought stress by reducing and eliminating ROS diffusion and production. Plants treated with AMF/PGPR counteract water deficit-induced oxidative stress by upregulating ROS-scavenging antioxidant compounds and antioxidant enzymatic activities. It is well known that plants protect against the damage caused by this oxidative stress by mechanisms that detoxify ROS which can be enzymatic (superoxide dismutase, catalase, ascorbate peroxidase, glutathione reductase, and monodehydroascorbate reductase) and non-enzymatic (flavanones, anthocyanins, carotenoids, and ascorbic acid). Our results showed a significant increase in POX and PPO in plants subjected to water deficit and inoculated with beneficial microbes and/or amended with compost than controls. Duo et al. (2018) revealed that nano-compost alone or combined with bacterial strains minimized the effects of drought stress by increasing antioxidant enzymes and decreasing MDA. Other studies suggested that drought tolerance is acquired by bacteria through the improvement of plant cell membrane stability and elasticity by activating the antioxidant defense system (Dimkpa et al., 2009; Gusain et al., 2015).

**FIGURE 9:**
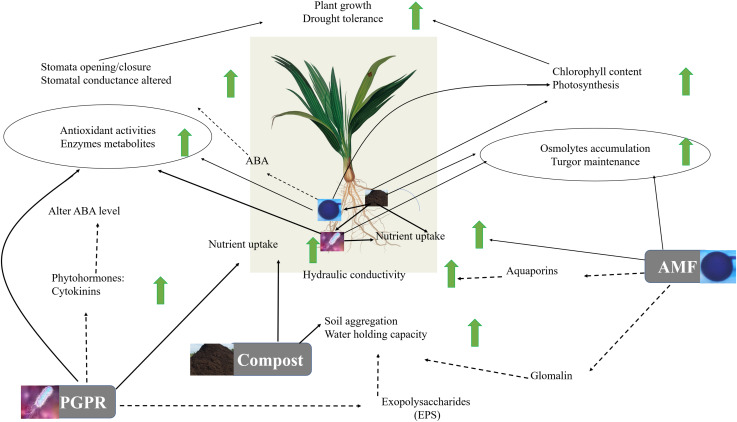
Suggested model for the regulatory network involved in date palm growth and tolerance to drought in response to compost, arbuscular mycorrhizal fungi (AMF) and plant growth promoting rhizobacteria (PGPR). According to this model, AMF colonization of a plant root permits the extension of hyphae extending into the surrounding soil, providing availability and storage of nutrients such as phosphorus and nitrogen for the plant. Also, AMF help to promote the synthesis of aquaporins which by changing the root hydraulic conductivity can enhance water uptake and water homeostasis maintenance under drought conditions. PGPR function as plant enhancer and facilitate the drought-exposed plants by improving nutrient uptake (N), water balance and osmoregulation through hormones (CKs and ABA)-mediating stomatal pores and regulating plant biochemical mechanisms (reducing the degradation of chlorophyll content and lipid peroxidation, increasing production of protein that reduces the damaging effect of ROS and can help maintain photosystem functionality under drought stress). Further, PGPR affect the EPS, allowing the increase of the water holding capacity. The compost functions as a soil conditioner in the process of decomposition and nutrient cycling (capture and delivery), which are driven by the activity of soil microorganisms affecting the soil microorganism activity (e.g., AMF and PGPR). The resulting changes in soil characteristics permit soil aggregation and enhance water holding capacity. Additionally, the plant–AMF/PGPR-compost associations act on physiological (increases in the photosynthetic pigments, and ABA-mediating higher stomatal conductance, permitting the increase of internal CO_2_ and photosynthetic capacity) and biochemical (accumulation of osmolytes and activation of antioxidant metabolites/activities allowing leaf osmotic adjustment, ROS scavenging, and alleviation of oxidative stresses) parameters. Solid lines represent the analyses carried out in this study. Dashed lines indicate mechanisms found in the literature.

The results highlight a physiological and biochemical switching mechanism in microbe association and provide additional confirmation of the hypothesis that, as illustrated in Figure 9, microbial association and compost operate at multiple (including photosynthesis machinery, antioxidant system, osmolytes biosynthesis, gene regulation) levels. Our study showed an improvement in the parameters studied in date palms growing in soil treated by the autochthonous biofertilizers mainly the consortium AMF2 alone and its combination with compost (C2) and/or PGPR (B2) under drought stress, especially AMF2+C2 and AMF2+C2+B2 treatments. This improvement in growth, mineral uptake, and physio-biochemical traits together with the decrease in MDA and H_2_O_2_ could be due to the synergy between AMF, compost, and PGPR: (1) the compost (C2) with low dose 5% allows good mycorrhizal infectivity, the presence of essential mineral elements such as N, P, and K for plant growth, and improves the soil quality by enhancing carbon, organic matter, and available P and N, (2) the native AMF hyphal structure might allow the uptake of water and nutrients needed by the plants and/or the changes in the level of phytohormones that participate in symbiosis. Furthermore, the stimulation of AMF symbiosis by root exudates could constitute an important source of organic carbon in the rhizosphere and a route of chemical communication between root plants and the fungi and (3) the PGPR (B2) could enhance phosphate solubilization resulting in increased phosphate available in the soil absorbed by plants through the production of organic acids and phosphatase. Furthermore, PGPR could modulate the tolerance of date palms via other mechanisms, yet to be elucidated, such as phytohormones (auxin, cytokinins), siderophores, and exopolysaccharide production.

Altogether, the general pattern that can be observed in the dataset is that an amendment of the soil reflects in better growth, in better soil properties and this also mirrors in stress related properties of photosynthesis; gas exchange or compound accumulation. It is worthy of note that the use of autochthonous biofertilizers (i.e., AMF2, C2, and B2) could constitute an original approach to improving the boost in growth and tolerance and may be a suitable combination for date palms in arid climates.

## Conclusion

Global agriculture will not only have to face the task of improving stress resistance and yields for food and biomass production but also that of reducing the dependence of producers on agrochemicals for a sustainable food system and environmental health. Therefore, the need to implement or revitalize eco-friendly technologies, such as compost and beneficial microbe-based biofertilization is of great importance for agriculture and the environment. Despite its enormous potential, the application of AMF, as yet, has not been fully adopted by farmers and the underlying mechanisms have not been sufficiently examined over recent decades. Moreover, since native AMF have demonstrated a great potential versus commercial isolates, in this work, it has been pointed out that native AMF, PGPR inoculation and compost overall produces positive outcomes on plant production, mainly owing to the several nutrition-related benefits that this class of soil beneficial microbes, symbionts and compost are able to provide to their host-plants. Furthermore, our data suggest that biofertilizers develop drought-adaptive strategies through the influence of plant mechanisms, such as photosynthetic machinery, better efficiency of PSII, root hydraulic conductivity, osmolyte accumulation, antioxidant enzyme production, higher membrane stability, and lower lipid peroxidation. Our findings are a first step toward encouraging farmers to autonomously produce their AMF inocula, starting from native soils. Further, this work makes biofertilization technology more likely to be affordable for farmers in harsh areas, and also those in developing countries for a sustainable crop growing system. For these reasons, the next significant step (an on-going study) toward the stable use of biofertilizers in agriculture and to better understand the potential effects of indigenous biofertilizers is carrying out field trials.

## Data Availability Statement

The datasets generated for this study are available on request to the corresponding author.

## Author Contributions

MA and MB designed and supervised the research. AM performed the experiments and carried out the analysis. AT, AR, AB, MA-E-M, RB-L, ST, and YA contributed analytic tools. MA, MB, and AM interpreted the data. MA, KO, MH, SS, and TM contributed to the conception and design of the work. AM and MB wrote the manuscript. AM, MB, and AM revised and finalized the manuscript. All authors read and approved the final version of the manuscript.

## Conflict of Interest

The authors declare that the research was conducted in the absence of any commercial or financial relationships that could be construed as a potential conflict of interest.

## Footnotes

1 http://www.fao.org

## Supplementary Material

Supplementary Figure 1 Phenotypic comparison in control date palm plants (non-amended, non-inoculated), and plants amended with composts (C1 or C2) and/or inoculated with arbuscular mycorrhizal fungi (AMF; exogenous AMF1 or native AMF2) or plant growth-promoting rhizobacteria (PGPR) strains (B1 or B2) under two water regimes [75 and 25% field capacity (FC)].

Supplementary Table 1A Result of multivariate analysis of variance MANOVA test for independent variables including Drought treatment, Composts (C1 and C2) amendment and exotic AMF (AMF1) and Bacteria (B1 and B2) inoculation and interaction among them.

Supplementary Table 1B Result of multivariate analysis of variance MANOVA test for independent variables including Drought treatment, Composts (C1 and C2) amendment and autochthonous AMF (AMF2) and Bacteria (B1 and B2) inoculation and interaction among them.

## References

[B1] Abbaspour H. Saeidi-Sar S. Afshari H. Abdel-Wahhab M. A. (2012). Tolerance of Mycorrhiza infected Pistachio (*Pistacia vera* L.) seedling to drought stress under glasshouse conditions. *J. Plant Physiol.* 169 704–709. 10.1016/j.jplph.2012.01.014 22418429

[B2] Abd El-Mageed T. A. El-Samnoudi I. M. Ibrahim A. E. A. M. Abd El Tawwab A. R. (2018). Compost and mulching modulates morphological, physiological responses and water use efficiency in *Sorghum bicolor* L. (Moench) under low moisture regime. *Agric. Water Manag.* 208 431–439. 10.1016/j.agwat.2018.06.042

[B3] Abd El-Mageed T. A. El-Sherif A. M. A. Abd El-Mageed S. A. Abdou N. M. (2019). A novel compost alleviate drought stress for sugar beet production grown in Cd-contaminated saline soil. *Agric. Water Manag.* 226:105831 10.1016/j.agwat.2019.105831

[B4] Abdel Latef A. A. H. Abu Alhmad M. F. Kordrostami M. Abo–Baker A. B. A. E. Zakir A. (2020). Inoculation with *Azospirillum lipoferum* or *Azotobacter chroococcum* reinforces maize growth by improving physiological activities under saline conditions. *J. Plant Growth Regul.* 39 1293–1306. 10.1007/s00344-020-10065-9

[B5] Abdel Latef A. A. H. Chaoxing H. (2011). Effect of arbuscular mycorrhizal fungi on growth, mineral nutrition, antioxidant enzymes activity and fruit yield of tomato grown under salinity stress. *Sci. Hortic.* 127 228–233. 10.1016/j.scienta.2010.09.020

[B6] Abdel Latef A. A. H. Hashem A. Rasool S. Abd_Allah E. F. Alqarawi A. A. Egamberdieva D. (2016). Arbuscular mycorrhizal symbiosis and abiotic stress in plants: a review. *J. Plant Biol.* 59 407–426. 10.1007/s12374-016-0237-7

[B7] Abdel Latef A. A. H. Mostofa M. G. Rahman M. M. Abdel-Farid I. B. Tran L.-S. P. (2019a). Extracts from yeast and carrot roots enhance maize performance under seawater-induced salt stress by altering physio-biochemical characteristics of stressed plants. *J. Plant Growth Regul.* 38 966–979. 10.1007/s00344-018-9906-8

[B8] Abdel Latef A. A. H. Kordrostami M. Zakir A. Zaki H. Saleh O. (2019b). Eustress with H_2_O_2_ facilitates plant growth by improving tolerance to salt stress in two wheat cultivars. *Plants* 8:303. 10.3390/plants8090303 31461870 PMC6783893

[B9] Adesemoye A. O. Torbert H. A. Kloepper J. W. (2009). Plant growth-promoting rhizobacteria allow reduced application rates of chemical fertilizers. *Micro Ecol.* 58 921–929. 10.1007/s00248-009-9531-y 19466478

[B10] Ahanger M. A. Tyagi S. R. Wani M. R. Ahmad P. (2014). “Drought tolerance: role of organic osmolytes, growth regulators, and mineral nutrients,” in *Physiological Mechanisms and Adaptation Strategies in Plants Under Changing Environment*, Vol. 1 eds Ahmad P. Wani M. R. (New York, NY: Springer), 25–55. 10.1007/978-1-4614-8591-9_2

[B11] Ait-El-Mokhtar M. Ben-Laouane R. Anli M. Boutasknit A. Wahbi S. Meddich A. (2019). Use of mycorrhizal fungi in improving tolerance of the date palm (*Phoenix dactylifera* L.) seedlings to salt stress. *Sci. Hortic.* 253 429–438. 10.1016/j.scienta.2019.04.066

[B12] Alikhani H. A. Saleh-Rastin N. Antoun H. (2006). Phosphate solubilization activity of rhizobia native to Iranian soils. *Plant Soil* 287 35–41. 10.1007/s11104-006-9059-6

[B13] Al-Karaki G. McMichael B. Zak J. (2004). Field response of wheat to arbuscular mycorrhizal fungi and drought stress. *Mycorrhiza* 14 263–269. 10.1007/s00572-003-0265-2 12942358

[B14] Al-Karaki G. N. (2000). Growth of mycorrhizal tomato and mineral acquisition under salt stress. *Mycorrhiza* 10 51–54. 10.1007/s005720000055

[B15] Al-Karaki G. N. (2006). Nursery inoculation of tomato with arbuscular mycorrhizal fungi and subsequent performance under irrigation with saline water. *Sci. Hortic.* 109 1–7. 10.1016/j.scienta.2006.02.019

[B16] Al-Karaki G. N. (2016). Application of mycorrhizal fungi in landscape turfgrass establishment under arid and semiarid environments. *AGROFOR* 1 154–161. 10.7251/agreng1602154a

[B17] Anli M. Symanczik S. El Abbassi A. Ait-El-Mokhtar M. Boutasknit A. Ben-Laouane R. (2020). Use of arbuscular mycorrhizal fungus *Rhizoglomus irregulare* and compost to improve growth and physiological responses of *Phoenix dactylifera* “Boufgouss”. *Plant Biosyst.* 1–14. 10.1080/11263504.2020.1779848

[B18] Arias E. Hodder A. J. Oihabi A. (2016). FAO support to date palm development around the world: 70 years of activity. *Emirates J. Food Agric.* 28 1–11. 10.9755/ejfa.2015-10-840

[B19] Arnon D. I. (1949). Copper enzymes in isolated chloroplasts. Polyphenoloxidase in *Beta vulgaris*. *Plant Physiol.* 24 1–15. 10.1104/pp.900074 16654194 PMC437905

[B20] Aubert G. (1978). *Méthodes d’analyses des sols*, 2ème Edn Marseille: Centre régional de Documentation Pédagogique, 191.

[B21] Augé R. M. Toler H. D. Saxton A. M. (2014). Arbuscular mycorrhizal symbiosis alters stomatal conductance of host plants more under drought than under amply watered conditions: a meta-analysis. *Mycorrhiza* 25 13–24. 10.1007/s00572-014-0585-4 24831020

[B22] Bano Q. Ilyas N. Bano A. Zafar N. Akram A. Hassan F. (2013). Effect of *Azospirillum* inoculation on maize (*Zea mays* L.) under drought stress. *Pakistan J. Bot.* 45 13–20.

[B23] Barje F. Meddich A. El Hajjouji H. El Asli A. Ait Baddi G. El Faiz A. (2016). Growth of date palm (*Phoenix dactylifera* L.) in composts of olive oil mill waste with organic household refuse. *Compost Sci. Util.* 24 273–280. 10.1080/1065657X.2016.1171738

[B24] Bárzana G. Aroca R. Ruiz-Lozano J. M. (2015). Localized and nonlocalized effects of arbuscular mycorrhizal symbiosis on accumulation of osmolytes and aquaporins and on antioxidant systems in maize plants subjected to total or partial root drying. *Plant Cell Environ.* 38 1613–1627. 10.1111/pce.12507 25630435

[B25] Baslam M. Goicoechea N. (2012). Water deficit improved the capacity of arbuscular mycorrhizal fungi (AMF) for inducing the accumulation of antioxidant compounds in lettuce leaves. *Mycorrhiza* 22 347–359. 10.1007/s00572-011-0408-9 21894519

[B26] Baslam M. Qaddoury A. Goicoechea N. (2014). Role of native and exotic mycorrhizal symbiosis to develop morphological, physiological and biochemical responses coping with water drought of date palm, *Phoenix dactylifera*. *Trees* 28 161–172. 10.1007/s00468-013-0939-0

[B27] Becklin K. M. Mullinix G. W. R. Ward J. K. (2016). Host plant physiology and mycorrhizal functionining shift across a glacial through future [CO_2_] gradient. *Plant Physiol.* 172 789–801. 10.1104/pp.16.00837 27573369 PMC5047097

[B28] Ben-Laouane R. Meddich A. Bechtaoui N. Oufdou K. Wahbi S. (2019). Effects of arbuscular mycorrhizal fungi and rhizobia symbiosis on the tolerance of *Medicago sativa* to salt stress. *Gesunde Pflanz.* 71 135–146. 10.1007/s10343-019-00461-x

[B29] Bompadre M. J. Silvani V. A. Bidondo L. F. Rios de Molina M. C. Colombo R. P. Pardo A. G. (2014). Arbuscular mycorrhizal fungi alleviate oxidative stress in pomegranate plants growing under different irrigation conditions. *Botany* 92 187–193. 10.1139/cjb-2013-0169

[B30] Bradford M. M. (1976). A rapid and sensitive method for the quantitation of microgram quantities of protein utilizing the principle of protein-dye binding. *Anal. Biochem.* 72 248–254. 10.1016/0003-2697(76)90527-3 942051

[B31] Casieri L. Ait Lahmidi N. Doidy J. Veneault-Fourrey C. Migeon A. Bonneau L. (2013). Biotrophic transportome in mutualistic plant-fungal interactions. *Mycorrhiza* 23 597–625. 23572325 10.1007/s00572-013-0496-9

[B32] Cavagnaro T. R. (2014). Impacts of compost application on the formation and functioning of arbuscular mycorrhizas. *Soil Biol. Biochem.* 78 38–44. 10.1016/j.soilbio.2014.07.007

[B33] Chao C. T. Krueger R. R. (2007). The date palm (*Phoenix dactylifera* L.)*:* Overview of biology, uses, and cultivation. *HortScience* 42 1077–1082. 10.21273/HORTSCI.42.5.1077

[B34] Chaumont F. Tyerman S. D. (2014). Aquaporins: highly regulated channels controlling plant water relations. *Plant Physiol.* 164 1600–1618. 10.1104/pp.113.233791 24449709 PMC3982727

[B35] Chitarra W. Pagliarani C. Maserti B. Lumini E. Siciliano I. Cascone P. (2016). Insights on the impact of arbuscular mycorrhizal symbiosis on tomato tolerance to water stress. *Plant Physiol.* 171 1009–1023. 10.1104/pp.16.00307 27208301 PMC4902612

[B36] De Pascale S. Rouphael Y. Colla G. (2017). Plant biostimulants: Innovative tool for enhancing plant nutrition in organic farming. *Eur. J. Hortic. Sci.* 82 277–285. 10.17660/eJHS.2017/82.6.2

[B37] Dimkpa C. Weinand T. Asch F. (2009). Plant-rhizobacteria interactions alleviate abiotic stress conditions. *Plant Cell Environ.* 32 1682–1694. 10.1111/j.1365-3040.2009.02028.x 19671096

[B38] Dubois M. Gilles K. A. Hamilton J. K. Rebers P. A. Smith F. (1956). Colorimetric method for determination of sugars and related substances. *Anal. Chem.* 28 350–356. 10.1021/ac60111a017

[B39] Duo L. A. Liu C. X. Zhao S. L. (2018). Alleviation of drought stress in turfgrass by the combined application of nano-compost and microbes from compost. *Russ. J. Plant Physiol.* 65 419–426. 10.1134/S102144371803010X

[B40] FAO (2018). *Drought characteristics and management in North Africa and the Near East.* Available online at: www.fao.org/3/CA0034EN/ca0034en.pdf

[B41] Fernández-Lizarazo J. C. Moreno-Fonseca L. P. (2016). Mechanisms for tolerance to water-deficit stress in plants inoculated with arbuscular mycorrhizal fungi. A review. *Agron. Colomb.* 34 179–189. 10.15446/agron.colomb.v34n2.55569

[B42] Frosi G. Barros V. A. Oliveira M. T. Santos M. Ramos D. G. Maia L. C. (2016). Symbiosis with AMF and leaf Pi supply increases water deficit tolerance of woody species from seasonal dry tropical forest. *J. Plant Physiol.* 207 84–93. 10.1016/j.jplph.2016.11.002 27875776

[B43] Grobelak A. Napora A. Kacprzak M. (2015). Using plant growth-promoting rhizobacteria (PGPR) to improve plant growth. *Ecol. Eng.* 84 22–28. 10.1016/j.ecoleng.2015.07.019

[B44] Gusain Y. S. Singh U. S. Sharma A. K. (2015). Bacterial mediated amelioration of drought stress in drought tolerant and susceptible cultivars of rice (*Oryza sativa* L.). *Afr. J. Biotechnol.* 14 764–773. 10.5897/AJB2015.14405

[B45] Hao Z. Xie W. Jiang X. Wu Z. Zhang X. Chen B. (2019). Arbuscular mycorrhizal fungus improves Rhizobium–glycyrrhiza seedling symbiosis under drought stress. *Agronomy* 9 1–12. 10.3390/agronomy9100572

[B46] Harley P. C. Loreto F. Di Marco G. Sharkey T. D. (1992). Theoretical considerations when estimating the mesophyll conductance to CO_2_ flux by the analysis of the response of photosynthesis to CO_2_. *Plant Physiol.* 98 1429–1436. 10.1104/pp.98.4.1429 16668811 PMC1080368

[B47] Hidri R. Barea J. M. Mahmoud O. M. Abdelly C. Azcón R. (2016). Impact of microbial inoculation on biomass accumulation by *Sulla carnosa* provenances, and in regulating nutrition, physiological and antioxidant activities of this species under non-saline and saline conditions. *J. Plant Physiol.* 201 28–41. 10.1016/j.jplph.2016.06.013 27393918

[B48] Hogekamp C. Arndt D. Pereira P. A. Becker J. D. Hohnjec N. Küster H. (2011). Laser microdissection unravels cell-type-specific transcription in arbuscular mycorrhizal roots, including CAAT-box transcription factor gene expression correlating with fungal contact and spread. *Plant Physiol.* 157 2023–2043. 10.1104/pp.111.186635 22034628 PMC3327204

[B49] Hori K. Wada A. Shibuta T. (1997). Changes in phenoloxidase activities of the galls on leaves of *Ulmus davidiana* formed by *Tetraneura fusiformis* (Homoptera: Eriosomatidae). *Appl. Entomol. Zool.* 32 365–371. 10.1303/aez.32.365

[B50] Jaleel C. A. Manivannan P. Wahid A. Farooq M. Al-Juburi H. J. Somasundaram R. (2009). Drought stress in plants: a review on morphological characteristics and pigments composition. *Int. J. Agric. Biol.* 11 100–105.

[B51] Javan Gholiloo M. Yarnia M. Ghorttapeh A. H. Farahvash F. Daneshian A. M. (2019). Evaluating effects of drought stress and bio-fertilizer on quantitative and qualitative traits of valerian (*Valeriana officinalis* L.). *J. Plant Nutr.* 42 1417–1429. 10.1080/01904167.2019.1628972

[B52] Júnior A. G. G. Pereira R. A. Sodré G. A. do Sacramneto C. K. Gross E. (2018). Inoculation with arbuscular micorrizhal fungi and organic compost from cocoa shell positively influence the growth and mineral nutrition of soursop plants (*Annona muricata* L.). *Rev. Bras. Frutic.* 40 1–11.

[B53] Khosravi Shakib A. Rezaei Nejad A. Khandan Mirkohi A. Kalate Jari S. (2019). Vermicompost and manure compost reduce water-deficit stress in pot marigold (*Calendula officinalis* L. cv. Candyman Orange). *Compost Sci. Util.* 27 61–68. 10.1080/1065657X.2019.1602489

[B54] Kohler J. Caravaca F. Azcón R. Díaz G. Roldán A. (2015). The combination of compost addition and arbuscular mycorrhizal inoculation produced positive and synergistic effects on the phytomanagement of a semiarid mine tailing. *Sci. Total Environ.* 514 42–48. 10.1016/j.scitotenv.2015.01.085 25659304

[B55] Kumar A. Patel J. S. Meena V. S. Srivastava R. (2019). Recent advances of PGPR based approaches for stress tolerance in plants for sustainable agriculture. *Biocatal. Agric. Biotechnol.* 20:101271 10.1016/j.bcab.2019.101271

[B56] Kumar R. K. Singh K. P. Raju D. V. S. (2015). Effect of different strains of arbuscular mycorrhizal fungi (AMF) on macro and micro nutrient uptake in micropropagated chrysanthemum plantlets. Vegetos-An Int. *J. Plant Res.* 28 47–54. 10.5958/2229-4473.2015.00036.1

[B57] Kumari B. Mallick M. A. Solanki M. K. Solanki A. C. Hora A. Guo W. (2019). “Plant growth promoting rhizobacteria (PGPR): Modern prospects for sustainable agriculture,” in *Plant Health Under Biotic Stress*, eds Ansari R. A. Mahmood I. (Singapore: Springer Singapore), 109–127. 10.1007/978-981-13-6040-4_6

[B58] Kwey M. M. Banze S. K. Mukalay J. B. (2015). Etude de cas sur l’impact des amendements organiques vis-à-vis de la salinité en culture de bananier. *Afrique Sci.* 11 152–160.

[B59] Lenoir I. Fontaine J. Lounès-Hadj Sahraoui A. (2016). Arbuscular mycorrhizal fungal responses to abiotic stresses: a review. *Phytochemistry* 123 4–15. 10.1016/j.phytochem.2016.01.002 26803396

[B60] Liao L. Dong T. Qiu X. Rong Y. Wang Z. Zhu J. (2019). Nitrogen nutrition is a key modulator of the sugar and organic acid content in citrus fruit. *PLoS One* 14:e0223356. 10.1371/journal.pone.0223356 31600253 PMC6786551

[B61] Liu X. Rezaei Rashti M. Dougall A. Esfandbod M. Van Zwieten L. Chen C. (2018). Subsoil application of compost improved sugarcane yield through enhanced supply and cycling of soil labile organic carbon and nitrogen in an acidic soil at tropical Australia. *Soil Tillage Res.* 180 73–81. 10.1016/j.still.2018.02.013

[B62] Luciens N. K. Yannick U. S. Yambayamba K. Michel M. M. Louis B. L. (2014). Amélioration des propriétés physiques et chimiques du sol sous l’apport combiné des biodéchets et des engrais minéraux et influence sur le comportement du maïs (*Zea mays* L. variété Unilu). *J. Appl. Biosci.* 6130 6121–6130.

[B63] Madhava Rao K. V. Sresty T. V. S. (2000). Antioxidative parameters in the seedlings of pigeonpea (*Cajanus cajan* (L.) Millspaugh) in response to Zn and Ni stresses. *Plant Sci.* 157 113–128. 10.1016/S0168-9452(00)00273-9 10940475

[B64] Maheshwari D. K. Kumar S. Maheshwari N. K. Patel D. Saraf M. (2012). “Nutrient availability and management in the rhizosphere by microorganisms,” in *Bacteria in Agrobiology: Stress Management*, ed. Maheshwari D. K. (Berlin: Springer), 301–326. 10.1007/978-3-642-23465-1

[B65] Meddich A. Ait El Mokhtar M. Bourzik W. Mitsui T. Baslam M. Hafidi M. (2018). “Optimizing growth and tolerance of date palm (*Phoenix dactylifera* L.) to drought, salinity, and vascular fusarium-induced wilt (*Fusarium oxysporum*) by application of arbuscular mycorrhizal fungi (AMF),” in *Root Biology*, eds Giri B. Prasad R. Varma A. (Cham: Springer), 239–258. 10.1007/978-3-319-75910-4

[B66] Meddich A. Boumezzough A. (2017). First detection of *Potosia opaca* larva attacks on *Phoenix dactylifera* and *P. canariensis* in Morocco: focus on pests control strategies and soil quality of prospected palm groves. *J. Entomol. Zool. Stud.* 5 984–991.

[B67] Meddich A. Elouaqoudi F. Khadra A. Bourzik W. (2016). Valorization of green and industrial waste by composting process. *J. Rev. Compos. Adv. Mater.* 26 451–469.

[B68] Meddich A. Jaiti F. Bourzik W. El Asli A. Hafidi M. (2015a). Use of mycorrhizal fungi as a strategy for improving the drought tolerance in date palm (*Phoenix dactylifera*). *Sci. Hortic.* 192 468–474. 10.1016/j.scienta.2015.06.024

[B69] Meddich A. Mézy M. Allain E. Toutain G. (2015b). Optimisation de la croissance et du developpement du palmier dattier en pepiniere par l’utilisation d’amendements biologiques, organiques et chimiques. *Eur. Sci. J.* 11 396–408.

[B70] Meddich A. Oihabi A. Jaiti F. Bourzik W. Hafidi M. (2015c). Rôle des champignons mycorhiziens arbusculaires dans la tolérance du palmier dattier (*Phoenix dactylifera*) à la fusariose vasculaire et au déficit hydrique. *Botany* 93 1–9.

[B71] Meddich A. Oufdou K. Boutasknit A. Raklami A. Tahiri A. Ben-Laouane R. (2019). “Use of organic and biological fertilizers as strategies to improve crop biomass, yields and physicochemical parameters of soil,” in *Nutrient Dynamics for Sustainable Crop Production*, ed. Meena R. S. (Singapore: Springer Singapore), 247–288. 10.1007/978-981-13-8660-2_9

[B72] Mehta C. M. Palni U. Franke-Whittle I. H. Sharma A. K. (2014). Compost: Its role, mechanism and impact on reducing soil-borne plant diseases. *Waste Manag.* 34 607–622. 10.1016/j.wasman.2013.11.012 24373678

[B73] Nadeem S. M. Ahmad M. Zahir Z. A. Javaid A. Ashraf M. (2014). The role of mycorrhizae and plant growth promoting rhizobacteria (PGPR) in improving crop productivity under stressful environments. *Biotechnol. Adv.* 32 429–448. 10.1016/j.biotechadv.2013.12.005 24380797

[B74] Nikitas C. Pocock R. Toleman I. Gilbert E. J. (2008). *The State of Composting and Biological Waste Treatment in the UK 2005/06.* Wellingborough: The composting Association.

[B75] Ning C. C. Gao P. D. Wang B. Q. Lin W. P. Jiang N. H. Cai K. Z. (2017). Impacts of chemical fertilizer reduction and organic amendments supplementation on soil nutrient, enzyme activity and heavy metal content. *J. Integr. Agric.* 16 1819–1831. 10.1016/S2095-3119(16)61476-4

[B76] Oihabi A. (1991). *Effet des endomycorhizes V.A sur la croissance et la nutrition minérale du palmier dattier.* Thèse de Doctorat d’Etat. Université Cadi Ayyad de Marrakech, Maroc/Univ. Bourgogne Dijon France, 117.

[B77] Olsen S. Sommers L. (1982). Methods of soil analysis. Part 2. Chemical and microbiological properties of phosphorus. *ASA Monograp.* 9 403–430.

[B78] Ortuño M. F. Lorente B. Hernández J. A. Sánchez-Blanco M. J. (2018). Mycorrhizal inoculation on compost substrate affects nutritional balance, water uptake and photosynthetic efficiency in *Cistus albidus* plants submitted to water stress. *Rev. Bras. Bot.* 41 299–310. 10.1007/s40415-018-0457-9

[B79] Padilla F. M. Peña-Fleitas M. T. Fernández M. D. Del Moral F. Thompson R. B. Gallardo M. (2017). Responses of soil properties, crop yield and root growth to improved irrigation and N fertilization, soil tillage and compost addition in a pepper crop. *Sci. Hortic.* 225 422–430. 10.1016/j.scienta.2017.07.035

[B80] Pagano M. C. (2014). “Drought stress and mycorrhizal plants,” in *Use of Microbes for the Alleviation of Soil Stress*, ed. Miransai M. (New York, NY: Springer), 97–110.

[B81] Paul M. J. Primavesi L. F. Jhurreea D. Zhang Y. (2008). Trehalose metabolism and signaling. *Annu. Rev. Plant Biol.* 59 417–441. 10.1146/annurev.arplant.59.032607.092945 18257709

[B82] Paymaneh Z. Sarcheshmehpour M. Bukovská P. Jansa J. (2019). Could indigenous arbuscular mycorrhizal communities be used to improve tolerance of pistachio to salinity and/or drought? *Symbiosis* 79 269–283. 10.1007/s13199-019-00645-z

[B83] Phillips J. M. Hayman D. S. (1970). Improved procedures for clearing roots and staining parasitic and vesicular-arbuscular mycorrhizal fungi for rapid assessment of infection. *Trans. Br. Mycol. Soc.* 55 158–161. 10.1016/S0007-1536(70)80110-3

[B84] Pieterse C. M. Zamioudis C. Berendsen R. L. Weller D. M. Van Wees S. C. Bakker P. A. (2014). Induced systemic resistance by beneficial microbes. *Annu. Rev. Phytopathol.* 52 347–375. 10.1146/annurev-phyto-082712-102340 24906124

[B85] Pozo M. J. Azcón-Aguilar C. (2007). Unraveling mycorrhiza-induced resistance. *Curr. Opin. Plant Biol*. 10 393–398. 10.1016/j.pbi.2007.05.004 17658291

[B86] Raklami A. Bechtaoui N. Tahiri A. Anli M. Meddich A. Oufdou K. (2019). Use of rhizobacteria and mycorrhizae consortium in the open field as a strategy for improving crop nutrition, productivity and soil fertility. *Front. Microbiol.* 10:1106. 10.3389/fmicb.2019.01106 31164880 PMC6536659

[B87] Rillig M. C. Wendt S. Antonovics J. Hempel S. Kohler J. Wehner J. (2014). Interactive effects of root endophytes and arbuscular mycorrhizal fungi on an experimental plant community. *Oecologia* 174 263–270. 10.1007/s00442-013-2759-8 23999946

[B88] Rodier J. (1984). *L’analyse de l’eau: eaux Naturelles, eaux résiduaires, eau de mer*, 7 ème Edn Paris: Dunod, 1365.

[B89] Sánchez-Romera B. Ruiz-Lozano J. M. Zamarreño ÁM. García-Mina J. M. Aroca R. (2016). Arbuscular mycorrhizal symbiosis and methyl jasmonate avoid the inhibition of root hydraulic conductivity caused by drought. *Mycorrhiza* 26 111–122. 10.1007/s00572-015-0650-7 26070449

[B90] Sandhya V. Ali S. Z. Grover M. Reddy G. Venkateswarlu B. (2010). Effect of plant growth promoting *Pseudomonas* spp. on compatible solutes, antioxidant status and plant growth of maize under drought stress. *Plant Growth Regul.* 62 21–30. 10.1007/s10725-010-9479-4

[B91] Sarwat M. Tuteja N. (2017). Hormonal signaling to control stomatal movement during drought stress. *Plant Gene* 11 143–153. 10.1016/j.plgene.2017.07.007

[B92] Sever K. Bogdan S. Franjiæ J. Škvorc Ž (2018). Nondestructive estimation of photosynthetic pigment concentrations in pedunculate oak (*Quercus robur* L.) leaves. *Sumar. List* 142 247–256.

[B93] Sghir F. Chliyeh M. Touati J. Mouria B. Touhami O.-A. Filali-Maltouf A. (2014). Effect of a dual inoculation with endomycorrhizae and *Trichoderma harzianum* on the growth of date palm seedlings. *Int. J. Pure Appl. Biosci.* 2 12–26.

[B94] Shaw R. G. Etterson J. R. (2012). Rapid climate change and the rate of adaptation: insight from experimental quantitative genetics. *New Phytol.* 195 752–765. 10.1111/j.1469-8137.2012.04230.x 22816320

[B95] Shen Z. Zhong S. Wang Y. Wang B. Mei X. Li R. (2013). Induced soil microbial suppression of banana fusarium wilt disease using compost and biofertilizers to improve yield and quality. *Eur. J. Soil Biol.* 57 1–8. 10.1016/j.ejsobi.2013.03.006

[B96] Strasser B. J. Strasser R. J. (1995). “Measuring fast fluorescence transients to address environmental questions: the JIP-test,” in *Photosynthesis: From Light to Biosphere*, ed. Mathis P. (Dordrecht: Springer Netherlands), 4869–4872. 10.1007/978-94-009-0173-5_1142

[B97] Symanczik S. Lehmann M. F. Wiemken A. Boller T. Courty P.-E. (2018). Effects of two contrasted arbuscular mycorrhizal fungal isolates on nutrient uptake by Sorghum bicolor under drought. *Mycorrhiza* 28 779–785. 10.1007/s00572-018-0853-9 30006910

[B98] Tartoura K. A. H. (2010). Alleviation of oxidative-stress induced by drought through application of compost in wheat (*Triticum aestivum* L.) plants. *Am. J. Agric. Environ. Sci.* 9 208–216.

[B99] Tartoura K. A. H. Youssef S. A. Tartoura E.-S. A. A. (2014). Compost alleviates the negative effects of salinity via up- regulation of antioxidants in *Solanum lycopersicum* L. plants. *Plant Growth Regul.* 74 299–310. 10.1007/s10725-014-9923-y

[B100] Tejera García N. A. Olivera M. Iribarne C. Lluch C. (2004). Partial purification and characterization of a non-specific acid phosphatase in leaves and root nodules of *Phaseolus vulgaris*. *Plant Physiol. Biochem.* 42 585–591. 10.1016/j.plaphy.2004.04.004 15331086

[B101] Trouvelot A. Kough J. L. Gianinazzi-Pearson V. (1986). Du taux de mycorhization VA d’un système radiculaire. Recherche de méthodes d’estimation ayant une signification fonctionnelle. *Mycorhizes Physiol. Génétique* 217–221. 10.1177/004057368303900411

[B102] Velikova V. Yordanov I. Edreva A. (2000). Oxidative stress and some antioxidant systems in acid rain-treated bean plants. *Plant Sci.* 151 59–66. 10.1016/s0168-9452(99)00197-1

[B103] Volpe V. Giovannetti M. Sun X. G. Fiorilli V. Bonfante P. (2016). The phosphate transporters LjPT4 and MtPT4 mediate early root responses to phosphate status in non mycorrhizal roots. *Plant Cell Environ.* 39 660–671. 10.1111/pce.12659 26476189

[B104] Vurukonda S. S. K. P. Vardharajula S. Shrivastava M. SkZ A. (2016). Enhancement of drought stress tolerance in crops by plant growth promoting rhizobacteria. *Microbiol. Res.* 184 13–24. 10.1016/j.micres.2015.12.003 26856449

[B105] Whitman E. (2019). A land without water: the scramble to stop Jordan from running dry. *Nature* 573 20–23. 10.1038/d41586-019-02600-w 31485066

[B106] Wu H. Xiang W. Chen L. Ouyang S. Xiao W. Li S. (2019). Soil phosphorus bioavailability and recycling increased with stand age in chinese fir plantations. *Ecosystems* 23 973–988. 10.1007/s10021-019-00450-1

[B107] Wu Q. Xia R. Hu Z. (2006). Effect of arbuscular mycorrhiza on the drought tolerance of *Poncirus trifoliata* seedlings. *Front. Forst. China* 1 100–104. 10.1007/s11461-005-0007-z

[B108] Wu Q. Zou Y. (2017). “Arbuscular mycorrhizal fungi and tolerance of drought stress in plants,” in *Arbuscular Mycorrhizas and Stress Tolerance of Plants*, ed. Wu Q.-S. (Singapore: Springer Singapore), 25–41. 10.1007/978-981-10-4115-0

[B109] Xie W. Hao Z. Zhou X. Jiang X. Xu L. Wu S. (2018). Arbuscular mycorrhiza facilitates the accumulation of glycyrrhizin and liquiritin in *Glycyrrhiza uralensis* under drought stress. *Mycorrhiza* 28 285–300. 10.1007/s00572-018-0827-y 29455337

[B110] Yadav K. Aggarwal A. Singh N. (2013). Arbuscular mycorrhizal fungi (AMF) induced acclimatization, growth enhancement and colchicine content of micropropagated *Gloriosa superba* L. plantlets. *Ind. Crops Prod.* 45 88–93. 10.1016/j.indcrop.2012.12.001

[B111] Yang W. Gu S. Xin Y. Bello A. Sun W. Xu X. (2018). Compost addition enhanced hyphal growth and sporulation of arbuscular mycorrhizal fungi without affecting their community composition in the soil. *Front. Microbiol.* 9:169. 10.3389/fmicb.2018.00169 29467752 PMC5808307

[B112] Yu Y. Y. Li S. M. Qiu J. P. Li J. G. Luo Y. M. Guo J. H. (2019). Combination of agricultural waste compost and biofertilizer improves yield and enhances the sustainability of a pepper field. *J. Plant Nutr. Soil Sci.* 182 560–569. 10.1002/jpln.201800223

[B113] Zhang F. Zou Y.-N. Wu Q.-S. (2018). Quantitative estimation of water uptake by mycorrhizal extraradical hyphae in citrus under drought stress. *Sci. Hortic.* 229 132–136. 10.1016/j.scienta.2017.10.038

[B114] Zhang Y. Zhong C. L. Chen Y. Chen Z. Jiang Q. B. Wu C. (2010). Improving drought tolerance of *Casuarina equisetifolia* seedlings by arbuscular mycorrhizas under glasshouse conditions. *New For.* 40 261–271. 10.1007/s11056-010-9198-8

[B115] Zou Y. N. Wu Q. S. (2011). Efficiencies of five arbuscular mycorrhizal fungi in alleviating salt stress of trifoliate orange. *Int. J. Agric. Biol.* 13 991–995.

