# Supplementary figures and images for: Biofertilizers as Strategies to Improve Photosynthetic Apparatus, Growth, and Drought Stress Tolerance in the Date Palm
Source: Front Plant Sci. 2020 Oct 23;11:516818. doi: 10.3389/fpls.2020.516818 (PMC7649861; doi:10.3389/fpls.2020.516818)

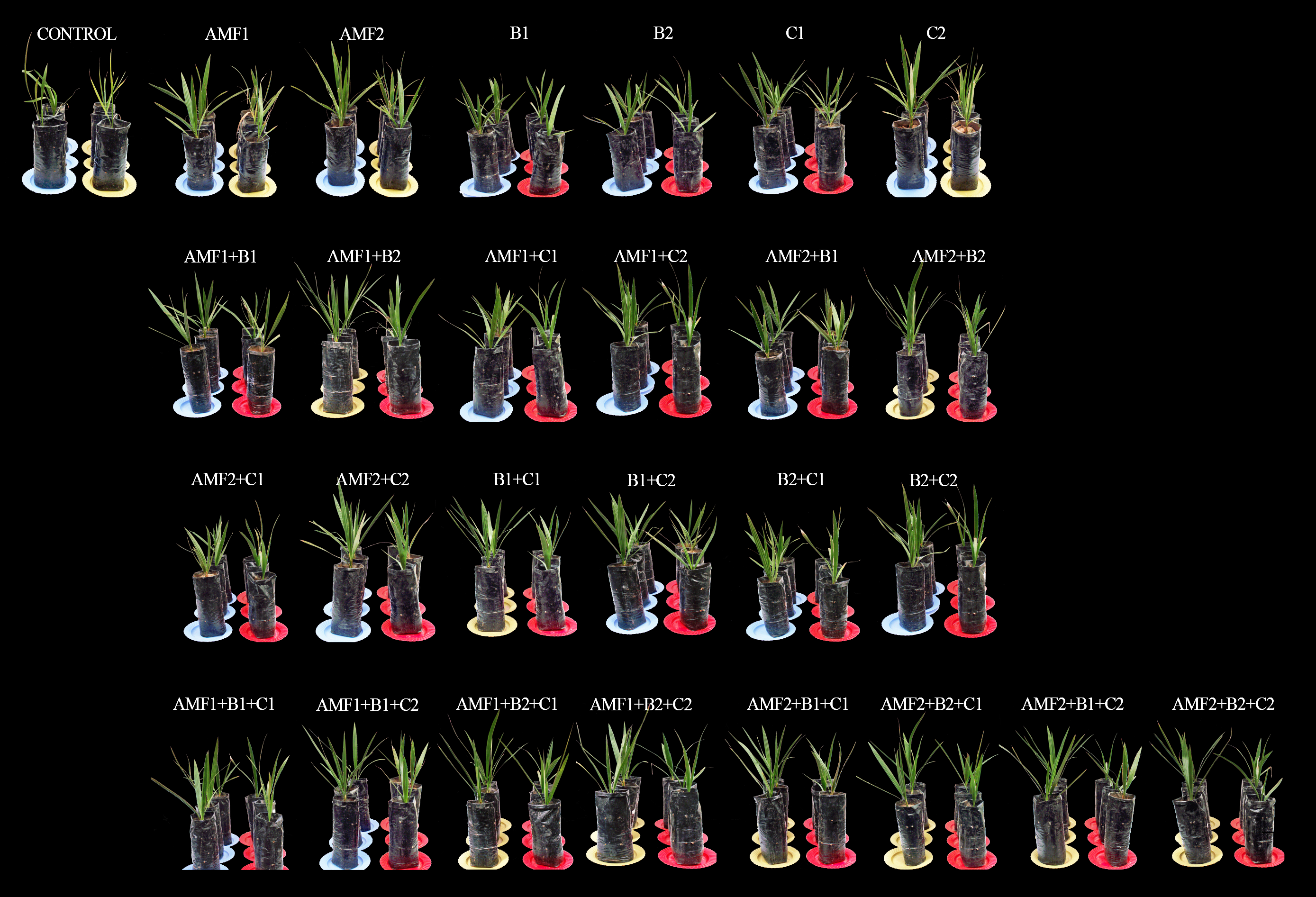

Supplement: Supplementary Figure 1 — Phenotypic comparison in control date palm plants (non-amended, non-inoculated), and plants amended with composts (C1 or C2) and/or inoculated with arbuscular mycorrhizal fungi (AMF; exogenous AMF1 or native AMF2) or plant growth-promoting rhizobacteria (PGPR) strains (B1 or B2) under two water regimes [75 and 25% field capacity (FC)]. [file Image_1.PNG]
