# Supplementary material for: Biofertilizers as Strategies to Improve Photosynthetic Apparatus, Growth, and Drought Stress Tolerance in the Date Palm
Source: Front Plant Sci. 2020 Oct 23;11:516818. doi: 10.3389/fpls.2020.516818 (PMC7649861; doi:10.3389/fpls.2020.516818)
Supplement: Supplementary Table 1A — Result of multivariate analysis of variance MANOVA test for independent variables including Drought treatment, Composts (C1 and C2) amendment and exotic AMF (AMF1) and Bacteria (B1 and B2) inoculation and interaction among them. [file Table_1.DOCX]

**Supplementary Table S1A:** Result of multivariate analysis of variance MANOVA test for independent variables including Drought treatment, Composts (C1 and C2) amendment and exotic AMF (AMF1) and Bacteria (B1 and B2) inoculation and interaction among them

| **Parameters** | **AMF1 (A1)** | **C1** | **C2** | **B1** | **B2** | **Drought (D)** | **A1 x C1** | **A1 x C2** | **A1 x B1** | **A1 x B2** | **A1 x D** | **A1 x C1 x B1** | **A1 x C1 x B2** | **A1 x C1 x D** | **A1 x C2 x B1** | **A1 x C2 x B2** | **A1 x C2 x D** | **C1 x B1** | **C1 x B2** |
| --- | --- | --- | --- | --- | --- | --- | --- | --- | --- | --- | --- | --- | --- | --- | --- | --- | --- | --- | --- |
| Mycorrhization frequency | *** | ns | ns | ns | ns | * | ns | ns | ns | ns | ns | ns | ns | ns | ns | ns | ns | ns | ns |
| Mycorrhization intensity | *** | ns | ns | ns | ns | ** | ns | ns | ns | ns | ns | ns | ns | ns | ns | ns | ns | ns | ns |
| Number of leaves | ns | ns | ns | ns | ns | *** | ns | ns | * | ns | ns | ** | ** | ns | ns | * | ns | ns | ns |
| Shoot height | ns | *** | ns | ns | ns | *** | * | *** | ns | ns | ns | * | ns | ns | ns | ns | ns | ns | ns |
| Root length | *** | * | ns | ns | ** | *** | ns | ns | ns | ns | ns | ns | ns | ns | ns | ns | ns | ns | ns |
| Leaf area | ns | * | ** | ns | ns | *** | * | * | ns | ns | ns | ns | ns | ns | ns | ns | ns | ns | ns |
| Total dry weight | ns | ns | ns | * | * | *** | ns | *** | ns | ns | ns | ns | ns | ns | ns | ns | ns | ns | ns |
| P (shoot) | ns | ns | ns | ns | ns | *** | ns | ns | ** | ns | ns | * | *** | ns | ns | ns | ns | *** | ns |
| N (shoot) | ns | ns | ns | ns | ns | *** | ns | *** | ns | ns | ns | ns | ns | ns | ns | ** | ns | * | ns |
| Leaf water potential | *** | ** | ns | ** | *** | *** | ns | ns | ** | ns | ns | *** | *** | ns | *** | *** | ns | * | ns |
| Stomatal conductance | ** | * | ** | ns | ns | *** | ** | ns | ns | ns | ns | ns | ns | ns | ns | ns | ns | ns | * |
| Fv/Fm | ns | ns | ns | ns | ns | *** | ns | ns | ns | ns | ns | *** | *** | ns | ns | * | ns | ns | ns |
| Chlorophyll a | ns | ns | * | *** | *** | *** | *** | *** | ** | ** | ** | ns | ns | ns | ns | ** | * | ns | ns |
| Chlorophyll b | ns | ns | ns | *** | *** | *** | ** | *** | * | ns | *** | ns | ns | ** | ns | ns | ns | ns | ns |
| Total Chlorophyll | ns | ns | ns | *** | *** | *** | *** | *** | ** | * | *** | ns | ns | * | ns | * | * | ns | ns |
| Carotenoid | ns | ns | ns | * | *** | *** | *** | ** | *** | *** | ** | ns | ns | ns | ns | ns | *** | ** | *** |
| P (soil) | ns | ns | ns | ** | ** | *** | ** | ns | *** | ns | ns | ** | ** | ns | ns | ns | ** | *** | ns |
| N (soil) | ns | ns | ns | * | * | *** | ns | ns | ns | ns | * | ns | ns | ns | ns | ns | ns | *** | ns |
| pH | * | * | ns | ns | ns | ns | ns | ns | ns | ns | ns | ** | ns | ns | *** | *** | ** | ns | ** |
| Electrical conductivity | ns | *** | *** | ns | ns | *** | ns | ns | ** | ns | ns | ns | ns | ns | ns | ns | ns | ns | ns |
| Total organic carbon | *** | *** | *** | * | ** | *** | ns | ** | ns | ns | ** | *** | *** | ns | *** | *** | ns | *** | * |
| Sugar | * | ns | ns | *** | ns | *** | *** | *** | ns | ns | ns | ns | * | ns | ns | ns | *** | * | *** |
| Proteins | *** | *** | *** | *** | *** | *** | ** | ns | ns | * | * | *** | *** | ns | *** | * | ns | ** | * |
| PPO | * | * | ns | *** | ns | *** | ** | ns | *** | *** | ** | * | ** | ns | ns | ns | * | *** | *** |
| POX | *** | ns | ns | ns | ns | *** | ns | ** | ** | ns | *** | ns | ns | ns | ** | *** | ns | * | * |
| MDA | ** | ns | ns | * | ns | *** | ** | ns | *** | *** | ns | ns | ns | ** | ns | ns | * | *** | *** |
| H_2_O_2_ | ns | ns | ns | ns | ns | *** | ns | ns | *** | *** | ns | ** | *** | ns | ns | *** | ** | *** | *** |

**Supplementary Table S1A:** (Continuation)

| **Parameters** | **C2 x B1** | **C2 x B2** | **A1 x B1 x D** | **A1 x B2 x D** | **A1 x C1 x B1 x D** | **A1 x C1 x B2 x D** | **A1 x C2 x B1 x D** | **A1 x C2 x B2 x D** | **C1 x B1 x D** | **C1 x B2 x D** | **C2 x B1 x D** | **C2 x B2 x D** | **C1 x D** | **C2 x D** | **B1 x D** | **B2 x D** |
| --- | --- | --- | --- | --- | --- | --- | --- | --- | --- | --- | --- | --- | --- | --- | --- | --- |
| Mycorrhization frequency | ns | ns | ns | ns | ns | ns | ns | ns | ns | ns | ns | ns | ns | ns | ns | ns |
| Mycorrhization intensity | ns | ns | ns | ns | ns | ns | ns | ns | ns | ns | ns | ns | ns | ns | ns | ns |
| Number of leaves | ns | *** | ns | ns | ns | ns | ns | ns | ns | ns | ns | ns | ns | ns | ns | ns |
| Shoot height | ns | ns | ns | ns | ns | ns | ns | ns | ns | ns | ns | ns | ns | ns | ns | ns |
| Root length | ** | ns | ns | ns | ns | ns | ns | ns | ns | ns | ns | ns | ns | ns | ns | ns |
| Leaf area | ns | ns | ns | ns | ns | ns | ns | ns | ns | ns | ns | ns | ns | ns | ns | ns |
| Total dry weight | ns | *** | ns | ns | ns | ns | ns | ns | ns | ns | ns | ns | ns | ns | ns | ns |
| P (shoot) | ns | ns | * | ns | * | ns | ns | ns | *** | ns | ns | ns | * | ns | ns | * |
| N (shoot) | ns | ** | ns | ns | ** | ns | ns | ns | ns | ns | ns | ns | ns | ns | ns | ns |
| Leaf water potential | ns | ns | ns | ns | * | ns | ns | ns | ns | ns | ns | ns | * | * | ns | ns |
| Stomatal conductance | ns | ns | ns | ns | ns | ns | ns | ns | ns | ns | ns | ns | ns | ns | ns | ns |
| Fv/Fm | ns | ns | ns | ns | ns | ns | ns | ns | ns | ns | ns | ns | ns | ns | ns | ns |
| Chlorophyll a | ns | ns | * | ns | ns | ns | * | ns | * | * | ** | * | ns | ns | *** | * |
| Chlorophyll b | ns | ns | ** | *** | ns | ns | ns | ns | ** | ** | *** | *** | * | * | *** | * |
| Total Chlorophyll | ns | ns | ** | ** | ns | ns | ns | ns | ** | ** | *** | *** | ns | ns | *** | * |
| Carotenoid | ns | ns | * | ns | ns | ns | ns | ns | ns | ns | ns | ns | ns | *** | ns | *** |
| P (soil) | ** | ns | ns | ns | * | ns | ns | ns | ** | ns | ns | ns | ** | ns | ** | * |
| N (soil) | *** | *** | ns | ns | ns | * | ns | ns | ns | ns | ns | ns | ns | ns | ns | ns |
| pH | ** | * | ns | ns | ns | ns | ns | ns | ns | ns | ns | ns | ns | * | ** | ns |
| Electrical conductivity | ns | * | ns | ns | ns | ns | ns | ns | ns | ns | ns | ns | ** | ** | ns | ns |
| Total organic carbon | *** | *** | * | ** | *** | ** | *** | * | ns | * | *** | *** | *** | *** | ** | *** |
| Sugar | ** | *** | ns | ns | ns | ns | ns | ns | ns | ns | ns | ns | ns | ns | ns | ns |
| Proteins | * | * | ns | ns | * | ns | ns | ns | ns | ns | ns | ns | ns | ** | * | * |
| PPO | *** | ** | ns | ns | ns | * | ns | ns | ns | * | * | * | ns | ns | ns | ns |
| POX | ns | ns | ** | ** | * | ns | * | ** | ns | ns | ns | ns | ns | ns | ns | ** |
| MDA | *** | *** | ns | ** | ns | ns | ns | ** | ns | *** | ns | * | ns | ns | ns | ns |
| H_2_O_2_ | ns | ns | ** | ns | ns | ns | ns | ns | ns | ns | ns | * | ns | *** | * | ** |

**Supplementary Table S1B:** Result of multivariate analysis of variance MANOVA test for independent variables including Drought treatment, Composts (C1 and C2) amendment and autochthonous AMF (AMF2) and Bacteria (B1 and B2) inoculation and interaction among them

| **Parameters** | **AMF2 (A2)** | **C1** | **C2** | **B1** | **B2** | **Drought (D)** | **A2 x C1** | **A2 x C2** | **A2 x B1** | **A2 x B2** | **A2 x D** | **A2 x C1 x B1** | **A2 x C1 x B2** | **A2 x C1 x D** | **A2 x C2 x B1** | **A2 x C2 x B2** | **A2 x C2 x D** | **C1 x B1** | **C1 x B2** |
| --- | --- | --- | --- | --- | --- | --- | --- | --- | --- | --- | --- | --- | --- | --- | --- | --- | --- | --- | --- |
| Mycorrhization frequency | *** | ns | ns | ns | ns | ** | ns | ns | ns | ns | ns | ns | ns | ns | ns | ns | ns | ns | ns |
| Mycorrhization intensity | *** | ns | ns | ns | ns | ** | ns | ns | ns | ns | ns | ns | ns | ns | ns | ns | ns | ns | ns |
| Number of leaves | ns | ns | ns | ns | ns | *** | ns | * | ns | ns | ns | ns | * | ns | ns | ns | ns | ns | ns |
| Shoot height | ** | *** | ** | ns | ns | *** | ns | ns | ns | ns | ns | ns | ns | ns | ns | ns | ns | ** | ns |
| Root length | ns | ns | ns | ns | *** | *** | * | * | ns | ns | ns | * | *** | ns | *** | ns | ns | ns | ns |
| Leaf area | *** | ns | ** | ns | ns | *** | *** | *** | ns | ns | ns | ns | ns | ns | ns | ns | ns | ns | ns |
| Total dry weight | *** | ns | ns | * | * | *** | ns | * | ns | ns | ** | * | ** | ns | * | * | ** | ** | ns |
| P (shoot) | *** | ** | ns | ns | ** | *** | ns | ** | *** | ns | ** | ** | *** | ns | ** | *** | ns | *** | *** |
| N (shoot) | *** | ns | * | ns | ns | *** | ns | ns | ns | ns | * | * | ns | ns | ** | ns | ns | ** | ns |
| Leaf water potential | ns | ns | ns | ns | *** | *** | *** | ns | ** | ns | ns | ns | ns | ns | ns | ns | ns | ns | ns |
| Stomatal conductance | * | ** | ** | ns | ns | *** | ns | * | ns | ns | ns | ns | ** | ns | ns | * | ns | ns | ns |
| Fv/Fm | ns | ns | ns | ns | ns | *** | * | * | ns | ns | ns | ns | ns | ns | ns | ns | ns | ns | ns |
| Chlorophyll a | *** | ** | *** | *** | *** | *** | *** | * | ** | ** | ** | ns | *** | ns | ** | ** | *** | ns | ns |
| Chlorophyll b | *** | ns | * | *** | *** | *** | ** | ** | ** | *** | ns | * | ** | * | * | *** | *** | ns | ns |
| Total Chlorophyll | *** | * | ** | *** | *** | *** | *** | ** | ** | *** | ** | ** | *** | ** | ** | *** | *** | ns | ns |
| Carotenoid | *** | ns | ** | *** | *** | *** | ** | *** | ns | ns | *** | *** | *** | *** | ** | *** | *** | * | * |
| P (soil) | ** | ** | ns | ns | *** | *** | ns | ** | *** | ns | ns | * | *** | ns | * | *** | ns | *** | *** |
| N (soil) | *** | ns | ns | ns | ns | *** | ns | * | ns | ns | * | * | * | ns | ** | ns | ns | *** | ns |
| pH | * | ns | ns | ns | ns | ns | ns | ns | ns | ns | ** | ns | ** | ns | * | ns | ** | ns | ** |
| Electrical conductivity | *** | *** | *** | ** | ns | *** | * | ** | *** | ns | * | ns | ns | ns | * | ns | * | * | ns |
| Total organic carbon | ns | *** | *** | ns | *** | *** | *** | ** | ** | * | * | * | *** | ns | ns | ns | ns | *** | *** |
| Sugar | *** | *** | *** | *** | ns | *** | ** | ** | ns | * | ** | *** | ** | * | ns | ns | *** | ns | *** |
| Proteins | *** | *** | *** | *** | *** | *** | *** | ns | ns | ** | ** | ns | * | ns | ns | ** | ns | ns | ns |
| PPO | *** | ns | ** | ns | ns | *** | ** | *** | ** | ns | ns | *** | *** | ns | * | ns | *** | * | ns |
| POX | ns | ns | ** | ns | ns | *** | ns | *** | ns | ns | ns | ns | ns | ns | ns | ** | ** | ns | ns |
| MDA | ns | ns | ns | ns | ns | *** | ns | ns | ns | ns | ns | ** | ** | * | ns | ns | * | *** | *** |
| H_2_O_2_ | ns | ns | ns | ns | ns | *** | ns | ** | ns | * | ns | *** | *** | ns | ** | ns | *** | * | ns |

**Supplementary Table S1B:** (Continuation)

| **Parameters** | **C2 x B1** | **C2 x B2** | **A2 x B1 x D** | **A2 x B2 x D** | **A2 x C1 x B1 x D** | **A2 x C1 x B2 x D** | **A2 x C2 x B1 x D** | **A2 x C2 x B2 x D** | **C1 x B1 x D** | **C1 x B2 x D** | **C2 x B1 x D** | **C2 x B2 x D** | **C1 x D** | **C2 x D** | **B1 x D** | **B2 x D** |
| --- | --- | --- | --- | --- | --- | --- | --- | --- | --- | --- | --- | --- | --- | --- | --- | --- |
| Mycorrhization frequency | ns | ns | ns | ns | ns | ns | ns | ns | ns | ns | ns | ns | ns | ns | ns | ns |
| Mycorrhization intensity | ns | ns | ns | ns | ns | ns | ns | ns | ns | ns | ns | ns | ns | ns | ns | ns |
| Number of leaves | ns | *** | ns | ns | ns | ns | ns | ns | ns | ns | ns | ns | ns | ns | ns | ns |
| Shoot height | ns | ns | ns | ns | ns | ns | ns | ns | ns | ns | ns | ns | ns | ns | ns | ns |
| Root length | ** | ns | ns | ns | ns | ns | ns | ns | ns | ns | ns | ns | ns | ns | ns | ns |
| Leaf area | ns | ns | ns | ns | ns | ns | ns | ns | ns | ns | ns | ns | ns | ns | ns | ns |
| Total dry weight | ns | ** | ns | * | ns | ns | ns | ns | ns | * | ns | ns | ns | ns | ns | ns |
| P (shoot) | ** | ns | ** | ** | ns | *** | ns | ** | *** | *** | ns | ns | ** | ns | ns | *** |
| N (shoot) | ns | ** | ns | ns | ns | ns | ns | ns | ns | ns | ns | ns | ns | ns | ns | ns |
| Leaf water potential | ** | ** | ns | ns | ns | ns | ns | ns | ns | ns | ns | ns | ns | * | ns | ns |
| Stomatal conductance | ns | ns | ns | ns | ns | ns | ns | ns | ns | ns | ns | ns | ns | ns | ns | ns |
| Fv/Fm | ns | ns | ns | ns | ns | ns | ns | ns | ns | ns | ns | ns | ns | ns | ns | ns |
| Chlorophyll a | ns | ns | ns | *** | ns | ns | *** | * | * | * | *** | ** | ns | * | ** | ns |
| Chlorophyll b | ns | ns | ns | ** | ns | ** | ns | ns | ** | *** | *** | *** | *** | *** | *** | ns |
| Total Chlorophyll | ns | ns | ns | *** | ns | ** | * | ns | ** | *** | *** | *** | * | *** | *** | ns |
| Carotenoid | ns | ns | ns | ** | *** | ns | *** | ns | ** | * | ** | ns | ns | ns | ns | ** |
| P (soil) | *** | * | ** | ns | * | *** | ns | ns | * | * | ns | ns | ** | ns | * | *** |
| N (soil) | *** | *** | ns | ns | * | ns | ns | ns | ns | ns | ns | ns | ns | * | ns | ns |
| pH | ** | ** | ns | * | ns | ns | ns | ns | ns | ns | ns | ns | ns | ns | ns | * |
| Electrical conductivity | ** | *** | ns | ns | ns | ns | * | ns | ns | ** | ns | ns | *** | *** | ns | ns |
| Total organic carbon | *** | *** | * | *** | ns | *** | ns | ns | ns | *** | ns | * | *** | *** | ns | ns |
| Sugar | ns | *** | ** | ns | ns | ns | ns | ns | ns | ns | ns | ns | ** | *** | ns | ns |
| Proteins | ns | * | ns | ns | * | * | ns | ns | ns | ns | ns | ns | ns | ** | ** | * |
| PPO | *** | *** | *** | ns | ** | *** | ** | ** | ns | ns | * | ns | ns | ns | ns | ns |
| POX | * | * | ns | * | ns | ns | ns | ns | * | ns | ns | ns | ns | ns | ns | ns |
| MDA | *** | *** | ns | * | ns | ns | * | ** | ns | *** | ns | ns | ns | ns | ns | ns |
| H_2_O_2_ | ns | ns | ns | ** | ns | * | * | ns | ns | ns | ns | ** | ns | ** | *** | *** |
